# A Panel of Diverse Assays to Interrogate the Interaction between Glucokinase and Glucokinase Regulatory Protein, Two Vital Proteins in Human Disease

**DOI:** 10.1371/journal.pone.0089335

**Published:** 2014-02-19

**Authors:** Matthew G. Rees, Mindy I. Davis, Min Shen, Steve Titus, Anne Raimondo, Amy Barrett, Anna L. Gloyn, Francis S. Collins, Anton Simeonov

**Affiliations:** 1 National Center for Advancing Translational Sciences, National Institutes of Health, Rockville, Maryland, United States of America; 2 National Human Genome Research Institute, National Institutes of Health, Bethesda, Maryland, United States of America; 3 Oxford Centre for Diabetes Endocrinology & Metabolism, University of Oxford, United Kingdom; 4 GE Healthcare, Life Sciences, Piscataway, New Jersey, United States of America; 5 NIHR Oxford Biomedical Research Centre, ORH Trust, OCDEM, Churchill Hospital, Oxford, United Kingdom; Albert-Ludwigs-University, Germany

## Abstract

Recent genetic and clinical evidence has implicated glucokinase regulatory protein (GKRP) in the pathogenesis of type 2 diabetes and related traits. The primary role of GKRP is to bind and inhibit hepatic glucokinase (GCK), a critically important protein in human health and disease that exerts a significant degree of control over glucose metabolism. As activation of GCK has been associated with improved glucose tolerance, perturbation of the GCK-GKRP interaction represents a potential therapeutic target for pharmacological modulation. Recent structural and kinetic advances are beginning to provide insight into the interaction of these two proteins. However, tools to comprehensively assess the GCK-GKRP interaction, particularly in the context of small molecules, would be a valuable resource. We therefore developed three robust and miniaturized assays for assessing the interaction between recombinant human GCK and GKRP: an HTRF assay, a diaphorase-coupled assay, and a luciferase-coupled assay. The assays are complementary, featuring distinct mechanisms of detection (luminescence, fluorescence, FRET). Two assays rely on GCK enzyme activity modulation by GKRP while the FRET-based assay measures the GCK-GKRP protein-protein interaction independent of GCK enzymatic substrates and activity. All three assays are scalable to low volumes in 1536-well plate format, with robust Z’ factors (>0.7). Finally, as GKRP sequesters GCK in the hepatocyte nucleus at low glucose concentrations, we explored cellular models of GCK localization and translocation. Previous findings from freshly isolated rat hepatocytes were confirmed in cryopreserved rat hepatocytes, and we further extended this study to cryopreserved human hepatocytes. Consistent with previous reports, there were several key differences between the rat and human systems, with our results suggesting that human hepatocytes can be used to interrogate GCK translocation in response to small molecules. The assay panel developed here should help direct future investigation of the GCK-GKRP interaction in these or other physiologically relevant human systems.

## Introduction

Glucokinase (GCK), the glucose sensor in hepatocytes and pancreatic β-cells, is the primary determinant of flux through glycolysis in both tissues and therefore plays a critical role in glucose homeostasis [1]. Hepatic GCK is also regulated at the post-transcriptional level by the predominantly liver-specific glucokinase regulatory protein (GKRP). GKRP is a competitive inhibitor of GCK with respect to glucose that localizes predominantly to the hepatocyte nucleus, sequestering GCK in the fasting state [2]–[4]. In rodent model systems, increases in plasma glucose concentrations result in rapid hepatic glucose uptake, binding of glucose to Gck, subsequent dissociation of Gck from Gkrp, and nuclear export of active Gck [5]–[7]. GKRP itself contains a single sugar-binding site capable of binding phosphate esters including fructose 1-phosphate (F1P) and fructose 6-phosphate (F6P). F6P, a glycolytic intermediate that accumulates in the fasting state, enhances the interaction between GCK and GKRP [8]. F1P, an intermediate in hepatic fructose breakdown, accumulates in the fructose-fed state and disrupts the GCK–GKRP interaction [9]. Accordingly, competitive binding of F1P or F6P to GKRP reinforces the glucose-dependent activity of hepatic GCK.

The clinical relevance of mutations in *GCK* has long been appreciated. Heterozygous inactivating *GCK* mutations cause maturity-onset diabetes of the young (MODY), homozygous or compound heterozygous inactivating mutations cause permanent neonatal diabetes mellitus (PNDM), and heterozygous activating mutations cause Congenital Hyperinsulinism (CHI) [10]. In the last few years, a potential role for GKRP in the context of human disease has also emerged. A common variant within the *GCKR* gene, which encodes GKRP, is reproducibly associated with inverse risk of type 2 diabetes (T2D) and lipid traits such as plasma cholesterol and triglyceride levels, and rare coding variants within *GCKR* show association with hypertriglyceridemia and diabetes-related phenotypes [11]–[13].

The observation that *GCK* activating mutations lead to enhanced insulin secretion and lower blood glucose levels stimulated interest in the activation of GCK as a potential therapy in the hyperglycemic context of T2D, and the subsequent discovery of small molecules that enhance GCK enzyme activity (glucokinase activators; GKAs) [14]. Ensuing efforts have led to the development of diverse GKAs with differing kinetic and pharmacokinetic properties. More than 100 GKA patents have been filed, and a multitude of clinical trials are on-going [15].

While GKAs have shown efficacy in lowering blood glucose levels and enhancing insulin secretion in animal models and short-term human trials, the potential risks of GCK activation by pharmacological modulators have been well documented. However, *in vivo* studies have shown GKAs are effective in activating GCK in both the liver and β-cells, making it difficult to interpret beneficial and/or detrimental tissue-specific effects of GCK activation in isolation. In β-cells, chronic GCK activation may lead to uncontrolled insulin secretion, causing episodes of hypoglycemia. Hypoglycemic episodes have been observed in individuals with *GCK* activating mutations, in animal models treated with GKAs, and in human studies of individuals with T2D treated with GKAs, particularly at higher doses [16], [17]. In the liver, GCK activation enhances hepatic glucose uptake and disposal, but could decrease circulating glucose levels at the expense of increased hepatic lipid biosynthesis. An increase in hepatic triglyceride content has been demonstrated during both short-term and long-term GKA treatment in rodent models [18], and plasma triglyceride concentrations have been shown to increase in at least one study in humans after 14 weeks of GKA treatment [17]. This is consistent with genetic associations with *GCKR*, but altered lipid profiles have not been reported in individuals with CHI due to activating *GCK* mutations [19], [20].

Development of small molecule probes specific to hepatic GCK could lead to a more complete understanding of the potential risks and benefits of activating GCK in the liver. While there have been efforts to develop liver-targeted activators of GCK by targeting liver-specific uptake mechanisms [21]–[23], additional insight could be gained by interrogating the interaction of GCK with the liver-specific GKRP, as GKRP does not appear to be functionally expressed in β-cells [24]. Additionally, as the transcriptional regulation of *GCK* and *GCKR* differ, identification of probes that specifically target GKRP may provide insight into metabolic features distinct from GCK activation. For example, there is evidence that murine *Gckr* is specifically up-regulated in hyperglycemic contexts, while *Gck* is down-regulated [25]. Recent kinetic and structural studies are beginning to provide invaluable insight into the interaction between these two critical proteins [26]–[29]. To provide further tools to probe the interaction between GCK and GKRP, we developed three recombinant protein-based assays using human GCK and GKRP, including a FRET-based protein-protein interaction assay that does not require GCK enzyme activity. In light of the potential challenges associated with identifying small-molecule inhibitors of protein-protein interactions, we validated each assay using known modulators of both GCK (GKAs, glucose) and GKRP (F1P and sorbitol 6-phosphate, S6P, an open-chain analogue of F6P) that modify the GCK-GKRP interaction interface, providing support for the feasibility of this approach [30]–[33]. The assays were miniaturized to single-digit microliter volumes to enable 1536-well high-throughput screening. We also investigated the GCK-GKRP interaction in a cellular context. While previous studies have demonstrated that the subcellular localization of rat Gck and Gkrp can be quantitatively measured through the use of high-content imaging technologies, such studies have yet to be extended to human model systems. Importantly, due to emerging literature highlighting the differences between rodent and human GKRP [3], [24], [34], we analyzed the GCK-GKRP interaction in both rat hepatocytes and primary human hepatocytes.

## Materials and Methods

### Recombinant Protein Production, Purification, and Initial Kinetic Characterization

Recombinant glutathione-S-transferase (GST)-tagged human GCK and FLAG-tagged GKRP proteins were prepared as described previously [24], [35]. Purity and concentration were measured by the Agilent 230 Protein Kit (Agilent Technologies UK Ltd, Stockport, UK) and Bio-Rad Bradford Protein Assay (Bio-Rad Laboratories Ltd, Hemel Hempstead, UK), respectively. The glucose S_0.5_ and Hill coefficient for GCK were calculated using a luminescent assay measuring the production of ADP by GCK (ADP-Glo™ Kinase Assay, Promega Corporation, Madison, WI, USA; see below). Luminescence was measured at 18 glucose concentrations from 0.1 to 100 mM (two separate experiments; each n = 8 for each concentration). The reactions were carried out in white 1536-well plates in a 3 µl volume with ATP in tenfold excess (4 mM) of the previously described K_m_ for recombinant GST-GCK (0.4 mM) [35] and incubated for 45 minutes at room temperature. The estimated value for the glucose S_0.5_ across the two experiments was 7.5±0.8 mM (mean ± SD) and the Hill coefficient was 1.53±0.1 (mean ± SD; **Figure S1**), consistent with previously reported values (S_0.5_ = 7.5–10 mM; Hill coefficient = 1.6–1.7) [36].

The ATP K_m_ for GCK was determined by fluorescent monitoring of NADH oxidation using a dual-coupling system whereby generation of ADP by GCK was coupled to oxidation of NADH via PK and LDH (**Figure S2A**) [37]. The final reaction contained 4 nM GCK, glucose in five-fold excess of the calculated glucose S_0.5_ (37.5 mM), and ATP concentrations ranging from 0 to 2 mM. The reaction was initiated by addition of ATP and read immediately, then every 10 seconds for 5 minutes. Each ATP concentration was tested in quadruplicate. The initial slopes (v_0_) for each concentration were determined by linear regression (GraphPad Prism, GraphPad Software, San Diego, CA, USA), and then v_0_ was plotted against ATP concentration (**Figure S2B**). As LDH is inhibited by its substrate pyruvate at concentrations above the pyruvate K_m_ [38], a nonlinear regression accounting for substrate inhibition was utilized to fit the curve and calculate the K_m_, which gave a value of 0.4±0.11 mM (mean ± SD). All kinetic screening assays were therefore carried out with 0.4 mM ATP and 5 mM glucose (concentrations at and below the calculated ATP K_m_ and glucose S_0.5_, respectively).

### Homogeneous Time-resolved Fluorescence (HTRF) Assays

Homogeneous time-resolved fluorescence (HTRF) technology (Cisbio Bioassays, Bedford, MA, USA) was utilized for antibody-based FRET measurement of the GCK-GKRP protein-protein interaction [39]. Recombinant human GST-GCK and FLAG-GKRP were mixed in an HTRF assay buffer containing 2 mM MgCl_2_, 25 mM KCl, 25 mM HEPES, 1 mM DTT, 0.1% Tween-20, and 0.025% BSA (all buffer components Sigma-Aldrich). The pH was adjusted to 7.1 by drop-wise addition of 10 N NaOH. Anti-FLAG and anti-GST FRET acceptor and donor-conjugated antibodies, respectively (anti-FLAG XL 665 and anti-GST K; both Cisbio Bioassays), were mixed in the manufacturer’s recommended HTRF reconstitution buffer (0.8 M KF and 50 mM phosphate, pH 7.0; both Sigma-Aldrich, St. Louis, MO, USA) and added to the HTRF assay mixture. 20 ng/well anti-FLAG XL 665 and 2.7 ng/well anti-GST K were added in 384-well plate format; these amounts were reduced to 0.7 ng/well and 0.0945 ng/well, respectively, in 1536-well plate format. For assays in 1536-well plate format, the two reaction mixes were dispensed using an Aurora Discovery BioRAPTR Flying Reagent Dispenser (BioRAPTR; Beckman Coulter, Inc., Brea, CA, USA) [40] (3 µl/well for protein-containing buffer; 1 µl/well for detection antibodies) into white 1536-well solid-bottom medium binding plates (Greiner Bio-One, Monroe, NC, USA). Plates were lidded and incubated at room-temperature for the times indicated before being read on an Envision 2104 Multilabel Plate Reader (PerkinElmer, Shelton, CT, USA) with excitation at 320 nm and dual-wavelength detection at the FRET donor wavelength (615 nm) and FRET acceptor wavelength (665 nm) with a D400/D630 mirror and a 90 µS delay. The signal ratio was calculated by dividing the signal from channel 1 (665 nm) by channel 2 (615 nm). For the LOPAC^1280^ library screen, column 1 was all components with DMSO (maximum signal) and column 3 was no GKRP with DMSO (minimum signal). These two control columns were used for data normalization. The final HTS protocol is in Table S1, and the data have been deposited in PubChem (AID 743207).

### HTRF Counter-assay

An HTRF control assay was developed in 1536-well plate format to identify compounds that affected HTRF detection by mechanisms other than specific modulation of the GCK-GKRP interaction. The final assay conditions for this counter-assay were identical to those for the HTRF assay above excluding GCK and GKRP, but with replacement of 0.0945 ng/well anti-GST K with the same amount of a europium-conjugated rabbit anti-mouse IgG antibody (Cisbio Bioassays). Negative control wells included HTRF reconstitution buffer with the anti-IgG antibody but excluded the anti-FLAG XL 665 antibody.

### Diaphorase-coupled Kinetic Assays

The glucose 6-phosphate dehydrogenase (G6PDH)-coupled kinetic assay described previously [24] was adapted for high-throughput screening by dispensing 2 µl diaphorase-coupled substrate assay buffer (pH 7.1) to give final reaction conditions of 25 mM HEPES, 25 mM KCl, 2 mM MgCl_2_, 1.4 mM 2-mercaptoethanol, 0.025% BSA, 0.01% Tween-20, 0.15 mM NADP^+^, 5 mM glucose, 0.4 mM ATP, 4 U/ml G6PDH, and 0.1 mM resazurin (all Sigma-Aldrich, except ATP which was from Promega) into black 1536-well medium binding plates (Greiner Bio-One) using a BioRAPTR. The reaction was then initiated by addition of 2 µl of diaphorase-coupled enzyme assay buffer containing indicated concentrations of GST-GCK, FLAG-GKRP, and diaphorase (Sigma-Aldrich; final concentration 0.1 mg/ml). Reaction progress was followed by quantitation of the fluorescent product resorufin generated from resazurin with excitation at 525 nm and emission at 590 nm using a ViewLux Microplate Imager. Plates were read every minute for 30 minutes. The coupling enzyme diaphorase was shown to be in excess by demonstration of linearity between NADPH concentration and fluorescence at concentrations up to 0.1 mM (i.e., 100% conversion of 0.1 mM resazurin) in **Figure S3A**. Similarly, G6PDH was shown to be in excess by demonstration of linearity between G6P concentration and NADPH fluorescence at concentrations up to 0.15 mM, the equivalent of 100% conversion of 0.15 mM NADP^+^ (**Figure S3B**). For the LOPAC^1280^ library screen, column 1 was all components with DMSO (maximum signal) and column 3 was no GCK with DMSO (minimum signal). These two control columns were used for data normalization. The final HTS protocol is in Table S2, and the data have been deposited in PubChem (AID 743205).

### Bioluminescence Assays

A luminescence-based assay was developed for detection of ADP generation by recombinant GST-GCK (ADP-Glo™ Kinase Assay; Promega). Final reaction conditions included 25 mM HEPES, 25 mM KCl, 2 mM MgCl_2_, 1 mM DTT, 0.025% BSA, 0.01% Tween-20, 5 mM glucose, and concentrations of GST-GCK and FLAG-GKRP as indicated, and the reaction was initiated by addition of ATP to a final concentration of 0.4 mM. The reaction was terminated and luminescence generated using ADP-Glo™ Reagent and Kinase Detection Reagent (both Promega). In 1536-well plate format, luminescence enzyme assay buffer was dispensed (2 µl/well) using a BioRAPTR into white 1536-well medium binding plates and initiated by addition of 1 µl 1.2 mM ATP. After lidded incubation for the times indicated at room temperature, 2.5 µl of ADP-Glo™ Reagent was added to each well and plates were incubated with lidding for 60 minutes at room temperature. Following 5 µl addition per well of Kinase Detection Reagent, plates were incubated with lidding for a further 30 minutes at room temperature before luminescent detection using a ViewLux Microplate Imager (PerkinElmer). For the LOPAC^1280^ library screen, column 1 was all components with DMSO (maximum signal) and column 3 was no GCK with DMSO (minimum signal). These two control columns were used for data normalization. The final HTS protocol is in Table S3, and the data have been deposited in PubChem (AID 743206).

### Small Molecule Controls

The specificity of all assays was validated by testing an 11-point (half-log dilution) concentration range of the known reaction modulators S6P, F1P, glucose (all Sigma-Aldrich), the GKA 2-Amino-5-(4-methyl-4H-(1,2,4)-triazole-3-yl-sulfanyl)-N-(4-methyl-thiazole-2-yl)benzamide (GKA-EMD) from EMD Millipore Inc. (Billerica, MA, USA), or the GKA (R)-3-Cyclopentyl-2-(4-methanesulfonyl-phenyl)-N-thiazol-2-yl-propionamide (GKA-Axon) from Axon Medchem (Groningen, The Netherlands). S6P, F1P, and glucose were dissolved in water; GKAs were dissolved in DMSO. For validation experiments with the HTRF counter-assay, suramin sodium salt and aurintricarboxylic acid (Sigma-Aldrich) were dissolved in DMSO and tested as 11-point titrations in the GCK-GKRP HTRF assay and IgG HTRF counter-assay.

### Compound Library and Screening

The Library of Pharmacologically Active Compounds (LOPAC^1280^, Sigma-Aldrich) was plated in 1536-well format with an Evolution P3 liquid dispenser (PerkinElmer) in columns 5–48 of 1536-well clear polypropylene compound plates (Greiner Bio-One). Five stock concentrations were tested: 10 mM, 2 mM, 400 µM, 80 µM, and 3.2 µM. General compound library preparation in 1536-well format, compound storage, and further details of compound management have been described previously [41].

1536-well control compound plates contained either DMSO or varying concentrations of GKA-EMD in DMSO in columns 1–4 added using a Cybi-Well (CyBio, Jena, Germany). Column 4 contained 26 mM GKA-EMD (n = 16 wells) while column 2 contained a 1.5-fold 11-point serial dilution of GKA-EMD (n = 2 for each concentration, starting concentration 26 mM); all other wells contained DMSO. For all screening experiments, 23 nl from control plates or compound plates were pin-transferred into each well of the 1536-well plate using a Kalypsys PinTool equipped with a 1536-pin head (Wako Chemicals USA, Richmond, VA, USA) [42]. Immediately following compound addition, a pre-read was taken utilizing the appropriate detection wavelengths to detect compound autofluorescence using a ViewLux Microplate Imager (PerkinElmer).

### Data Analysis

Assay performance was measured by metrics including the Z’ score, signal-to-background ratio (S/B), and percent variance (%CV). For each assay a maximum signal and a minimum signal control column were utilized (described above under each assay type) and an average of each column was calculated and used in the three equations. S/B was calculated by (maximum signal)/(minimum signal). %CV was calculated by (standard deviation of compound area)/(mean compound area). Z’ was calculated by 1−(3×(standard deviation of minimum signal + standard deviation of maximum signal))/(median of maximum signal − median of minimum signal). Data were normalized and compound concentration-response curves fit using algorithms described previously [43].

### Cryopreserved Primary Hepatocyte Culture

All hepatocytes were purchased from Bioreclamation In Vitro Technologies (BioreclamationIVT, Baltimore, MD, USA). Plateable cryopreserved pooled male Sprague-Dawley rat hepatocytes (lot number NPD) were thawed and plated (40,000 cells/well) in Biocoat black/clear collagen-coated 96-well plates (BD Biosciences, San Jose, CA, USA) in *InVitro*GRO™ CP Rat medium supplemented with Torpedo™ Antibiotic Mix (BioreclamationIVT). After hepatocyte attachment, media was changed to *InVitro*GRO™ HI Rat medium with Torpedo™ Antibiotic Mix. After 24 hours, cells were incubated for 12 hours in glucose-free DMEM containing 10% dialyzed FBS, 100 nM insulin, 10 nM dexamethasone, 2 mM L-glutamine, 50 U/ml penicillin, and 50 µg/ml streptomycin (all Invitrogen, Life Technologies Corporation, Carlsbad, CA, USA) before treatment for two hours with DMEM containing glucose and GKA-EMD to the concentrations indicated.

Plateable cryopreserved human hepatocytes (female, lot number TRZ; male, lot number FOS) were plated at 50,000 cells/well in Biocoat black/clear collagen-coated 96-well plates in *InVitro*GRO™ CP medium supplemented with Torpedo™ Antibiotic Mix. After hepatocyte attachment, media was changed to *InVitro*GRO™ HI medium with Torpedo™ Antibiotic Mix. After 24 hours, cells were incubated for 12 hours in glucose-free DMEM containing 10% dialyzed FBS, 100 nM insulin, 10 nM dexamethasone, 2 mM L-glutamine, 50 U/ml penicillin, and 50 µg/ml streptomycin (all Invitrogen) before treatment for two hours with DMEM containing glucose, sorbitol, and GKA-EMD to the concentrations indicated.

### Cell fixation and Immunofluorescence

Hepatocytes were fixed in 4% PFA for 15 minutes. PFA was then removed and cells were washed twice in Dulbecco’s phosphate-buffered saline (DPBS) containing 0.2% Triton-X and then permeabilized for 10 minutes with DPBS/0.2% Triton-X. Cells were then treated for 30 minutes with Odyssey protein blocking buffer (Li-cor Biosciences, Lincoln, NE, USA) before addition of a mouse monoclonal IgG_2a_ GKRP antibody [GCKR (A-8); Santa Cruz Biotechnology, Inc., Santa Cruz, CA, USA]. The antibody (200 µg/ml) was diluted (1∶50) in protein blocking buffer and incubated with cells for 120 minutes. Cells were then washed (4×5 minutes) in PBS/0.1% Tween-20 before addition of protein blocking buffer containing Alexa Fluor® 647 Goat Anti-Mouse IgG (H+L) (Invitrogen; 1∶500 dilution) and rabbit polyclonal GCK antibody [1∶30 dilution; GCK (H-88) 200 µg/ml; Santa Cruz Biotechnology]. After 60 minutes, cells were washed with PBS/0.1% Tween-20 (4×5 minutes). Following a 60-minute incubation with blocking buffer and goat anti-rabbit IgG-HRP (1∶500 dilution; sc-2004; Santa Cruz Biotechnology), cells were washed with PBS/0.1% Tween-20 (4×5 minutes). Fluorescent detection of the HRP-conjugated secondary antibody was enabled using a 10-minute incubation with fluorescein-conjugated tyramide from the Tyramide Signal Amplification kit (PerkinElmer). The tyramide solution was prepared in DMSO (Sigma-Aldrich) according to the manufacturer’s instructions. Cells were then washed (3×5 minutes) with PBS/0.1% Tween-20 before a final five-minute wash with PBS/0.1% Tween-20 containing 0.02 mg/ml Hoechst 33342 Trihydrochloride Trihydrate (Hoechst; Invitrogen) for visualization of nuclei. After Hoechst staining, all cells were then stored in 100 µl DPBS at 4**°**C. Control wells on each plate to account for non-specific background included primary antibodies only or secondary antibodies only, both in the presence and absence of fluorescein-conjugated tyramide.

### Image Analysis of Hepatocytes

After antibody staining and fixation, plates were imaged on an IN Cell 2000 (GE Healthcare, Piscataway, NJ, USA) using a 10×0.45 NA objective with standard DAPI (100 msec exposure), FITC (100 msec exposure), and Cy5 (2000 msec exposure) filter sets. Image analysis was conducted using the Multi Target Analysis algorithm in IN Cell Analyzer Workstation v 3.7.1. Nuclei (in the DAPI channel) with an average minimum size of 50 µm were segmented using the top hat segmentation method. The FITC channel was utilized to detect GCK-expressing cells with an average minimum size of 250 µm. These cells were segmented using the multi scale top hat segmentation method. GKRP-expressing cells were identified using the Cy5 signal and used as a reference to the FITC cell channel. For human hepatocytes, subpopulations of Cy5 positive and negative cells were generated based on the average intensity of the nuclear Cy5 signal; greater than 300 was considered positive. In the positive population, translocation events were characterized based on the ratio of nuclear to cell signal in the FITC channel; anything with a ratio of 1.2 or greater was considered to be translocation positive. Translocation concentration-response curves were fitted using nonlinear regression with the log(agonist) vs. response option in GraphPad Prism.

## Results

### Assay Development Strategy

**Figure 1:**
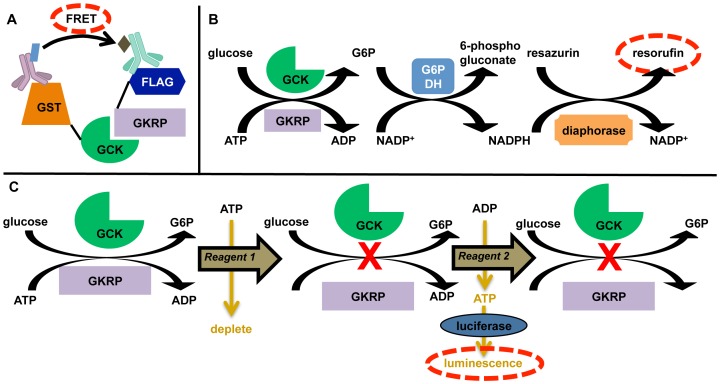
Parallel assay development strategy to interrogate recombinant GCK and GKRP. Human GCK and GKRP were affinity-purified using GST and FLAG tags, respectively. (A) FRET-based (HTRF) detection. Antibodies recognizing the affinity tags are conjugated to FRET donor and acceptor molecules. Excitation of the donor results in energy transfer (FRET; red dashed oval) to the acceptor only if the acceptor and donor are in close proximity. (B) Reaction scheme for G6PDH/diaphorase dual-coupled assay. The generation of the fluorescent product resorufin (red dashed oval) is measured as the reaction progresses in real time by excitation at 525 nm with emission at 590 nm. (C) Reaction scheme for coupling of ADP generation by GCK to luminescence-based detection. The GCK reaction is allowed to run for a set period of time, and the reaction is then terminated and a two-step reaction utilizes Ultra-Glo™ firefly luciferase to generate bioluminescence (red dashed oval). Reagent 1: ADP-Glo™ Reagent; Reagent 2: Kinase Detection Reagent.

A parallel assay development approach was undertaken to query multiple features of the GCK-GKRP interaction, including direct protein-protein binding, real-time monitoring of enzymatic activity, and kinetic endpoint measurements (**Figure 1**). Additionally, each assay was selected to utilize a different detection methodology, with the goal of providing a range of assays that would facilitate the differentiation of potential on-target small molecules from false-positive assay artifacts.

### Development of HTRF Assay

**Figure 2:**
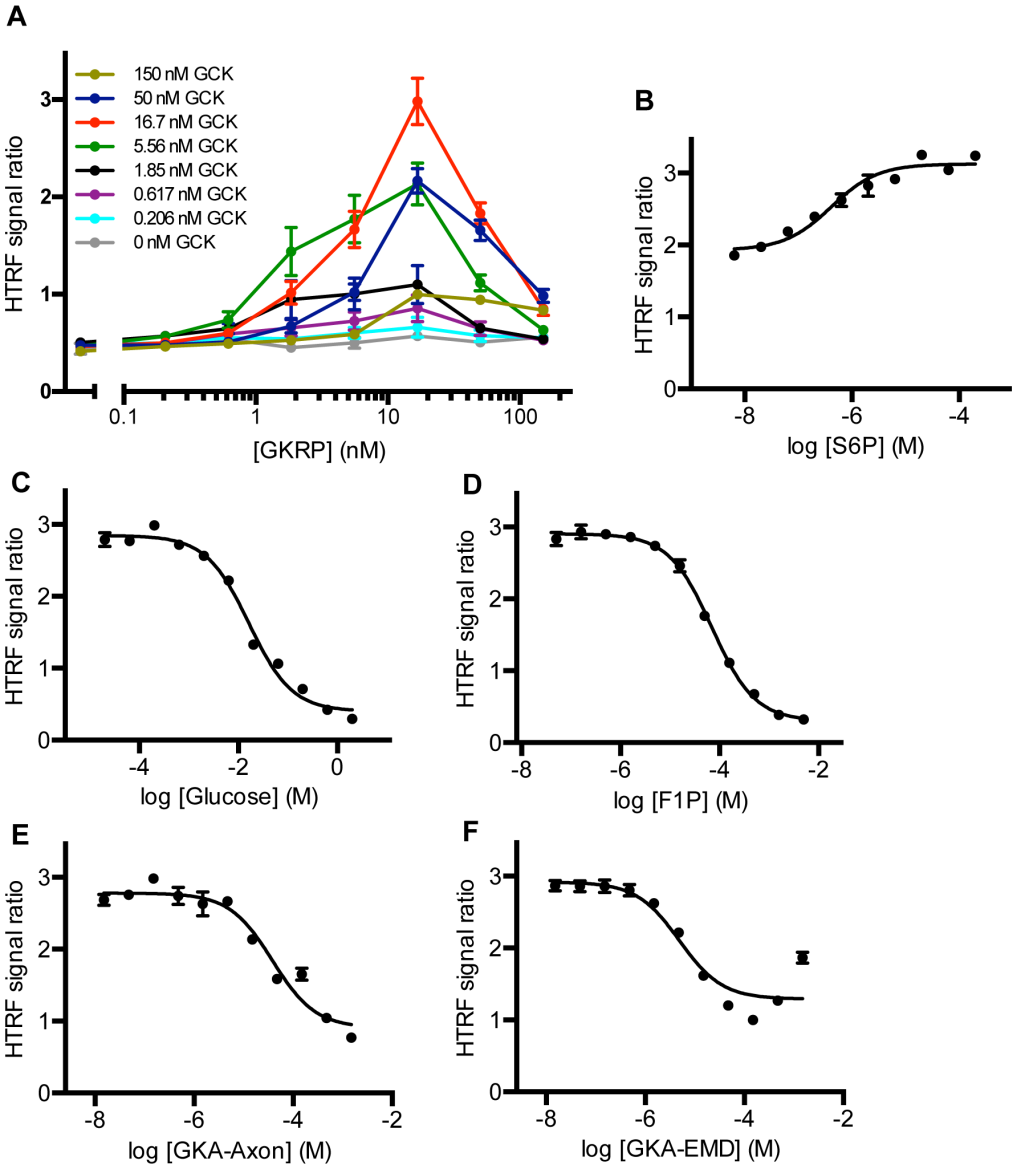
Optimization and validation of HTRF-based detection of the GCK-GKRP interaction. (A) Titration of GCK and GKRP. Results are presented as the ratio of the acceptor emission (665 nm) and donor emission (615 nm) for eight different GCK and GKRP concentrations measured at t = 60 minutes. Each data point is mean ± SEM for n = 6. (B) Effect of S6P on HTRF detection of the GCK-GKRP interaction. The experiment was run in the presence of 5 nM GCK and 5 nM GKRP. Values shown are mean ± SEM (n = 8 for each [S6P]). (C–F) Effect of known inhibitors glucose (C), F1P (D), and two GKAs (E–F) on HTRF detection of the GCK-GKRP interaction in the presence of 5 nM GCK, 5 nM GKRP, and 2 µM S6P. Values shown are mean ± SEM (n = 8 for each concentration).

An HTRF system [39] for detection of the GCK-GKRP interaction was utilized to recognize GST-tagged recombinant GCK with macrocyclic europium attached to an anti-GST antibody, and XL 665 attached to an anti-FLAG antibody was utilized to recognize FLAG-tagged recombinant GKRP, initially in 384-well-plate format (**Figure 1A**). The assay was validated using a range (0–150 nM) of GCK and GKRP concentrations at manufacturer-recommended detection antibody concentrations (**Figure 2A**). Fluorescence was measured immediately upon mixing, and every 30 minutes thereafter for a total of 240 minutes. The plate was covered and incubated at room temperature throughout the course of the experiment. The signal reached a maximum at t = 60 minutes and remained stable (Figure S4); the results at 60 minutes are presented in **Figure 2A**, and this incubation time was selected as the optimum for all future measurements.

The assay was miniaturized to 1536-well plate format and, concurrently, specificity of detection was validated by using known modulators of the GCK-GKRP interaction. S6P, an open-chain analogue of F6P, enhances the interaction of GCK and GKRP with higher potency than F6P, with a previously reported half-maximal effective concentration (EC_50_) of approximately 0.5 µM [34]. Protein concentrations of 5 nM GCK and 5 nM GKRP were selected to provide a window to detect enhancement of FRET signal and five concentrations of S6P were tested on either side of the predicted EC_50_ value using half-log dilutions. These reactions were carried out in a 4 µl final volume in a 1536-well plate and demonstrated that the reaction was scalable to 1536-well format with no appreciable loss of assay performance (**Figure 2B**). The signal ratio in the absence of S6P in this experiment (mean = 1.87) was similar to that observed in 384-well format at 5.56 nM of both proteins (mean = 1.77). The S6P EC_50_ value given from a nonlinear regression of these data was 0.4±0.08 µM (mean ± SD), in agreement with previous findings.

As S6P enhanced detection and is available commercially for a fraction of the cost of GKRP, subsequent experiments were run including S6P at its EC_80_ value (2 µM). The EC_80_ value represents a compromise between the desire for large enough signal enhancement and the need to maintain a binding level that is not too saturated so that it can be outcompeted by compounds of interest. Additionally, this approach enables detection of specific competitors of S6P, which would likely be acting on GKRP as desired, and not on GCK. Therefore, the effect of known inhibitors of the GCK-GKRP interaction, which should decrease the fluorescence signal, were tested at 5 nM GCK, 5 nM GKRP, and 2 µM S6P. Four compounds were tested at five half-log concentrations below and above their predicted half-maximal inhibitory concentration (IC_50_) values: glucose (predicted IC_50_ = 20 mM), F1P (50 µM), and two commercially available GKAs (15 µM). Both glucose and GKAs interfere with the binding of GKRP to GCK and decrease GKRP inhibition [30], [44], [45], while F1P binds to GKRP and is competitive with respect to S6P [46]. All four compounds appreciably inhibited the GCK-GKRP interaction with an associated decrease in the HTRF signal (glucose IC_50_ = 16±1.4 mM; F1P IC_50_ = 70±5.4 µM; GKA-Axon IC_50_ = 38±6.8 µM; GKA-EMD IC_50_ = 49±1.0 µM; all mean ± SD) (**Figure 2C–2F**). Therefore, concentrations of 5 nM GCK, 5 nM GKRP, and 2 µM S6P were suitable for detection of inhibition of the GCK-GKRP interaction via both GCK and GKRP and were chosen for subsequent high-throughput experiments.

**Figure 3:**
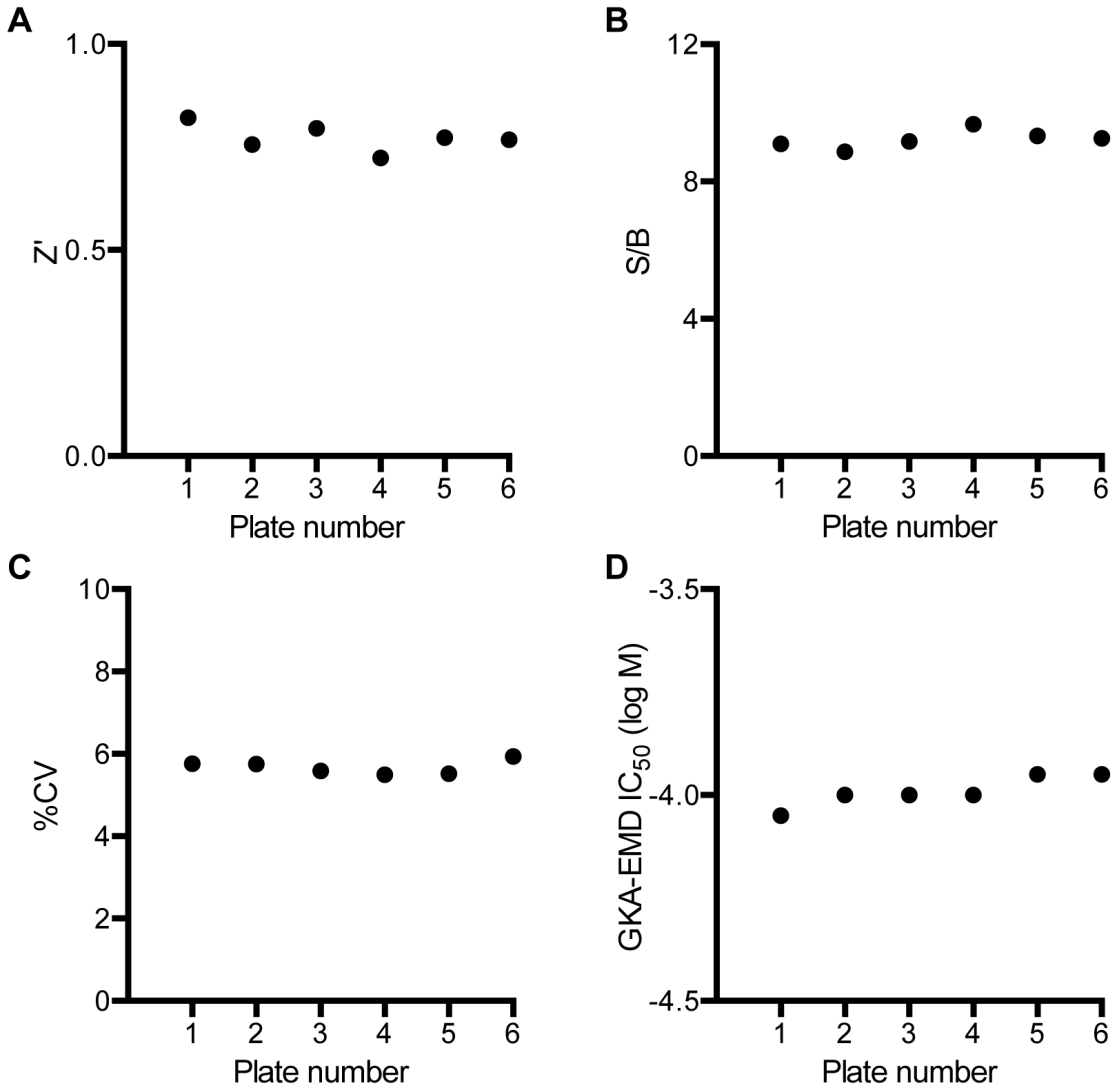
Quality metrics for the HTRF assay with the LOPAC^1280^ library in 1536-well plates. (A) Z’ factor, (B) signal/background, (C) % variance, and (D) calculated IC_50_ of the control compound GKA-EMD as a function of assay plate.

To evaluate the assay’s ability to detect small molecule modulators of the GCK-GKRP interaction, we next performed a screen of a small library of bioactive molecules, the LOPAC^1280^ collection, as a dilution series. Six source plates were tested, the first containing only DMSO as a vehicle control, and the remainder containing DMSO solutions of library compounds at five different concentrations to allow for generation of concentration response curves. Controls included GKA-EMD and all assay components without GKRP (negative control; n = 32 wells/plate). Compounds (23 nl) were pin-transferred into a 3 µl volume containing assay buffer, GCK, GKRP, and S6P; the HTRF anti-tag detection reagents were added (1 µl/well) following a pre-read to detect compound autofluorescence. The assay performed well across all plates (Z’ = 0.81±0.035; CV = 5.7±0.17; S/B = 9.2±0.27; mean ± SD across six plates) (**Figure 3**), and demonstrated consistent inhibition by the control compound GKA-EMD delivered as a titration within each screening plate. Additionally, there was no loss in assay performance upon overnight storage of the reagents on ice.

### Counter-assay to Identify Compounds Interfering with HTRF Detection

The HTRF-based assay was adapted to detect small molecules that interfere non-specifically with protein-protein interactions, with HTRF reaction components, or with detection conditions. A rabbit anti-mouse IgG antibody was utilized as the FRET donor in place of the GST K antibody, with the monoclonal mouse anti-FLAG XL 665 antibody (as above) utilized as the FRET acceptor. Accordingly, mixture of these two antibodies should result in donor-acceptor antibody interaction, and thus detectable FRET signal, in the absence of interference. True positives, such as F1P and S6P, should show no concentration-dependent change in HTRF signal in this control experiment (**Figure S5A**), while compounds that show fluorescent liabilities in either the donor or acceptor channels should result in a change in signal in this IgG-based assay. Such compounds can therefore be confirmed to be due to interference with the detection system (**Figure S5B**; suramin sodium salt IgG counter-assay IC_50_ = 0.25±0.07 µM; mean ± SD). Additionally, compounds without fluorescent liabilities but that show an effect in both the GCK-GKRP HTRF assay and this counter-assay can be identified as likely false positives (**Figure S5C**; ATA IgG counter-assay IC_50_ = 0.09±0.03 µM; mean ± SD).

### Diaphorase-coupled Kinetic Assay

**Figure 4:**
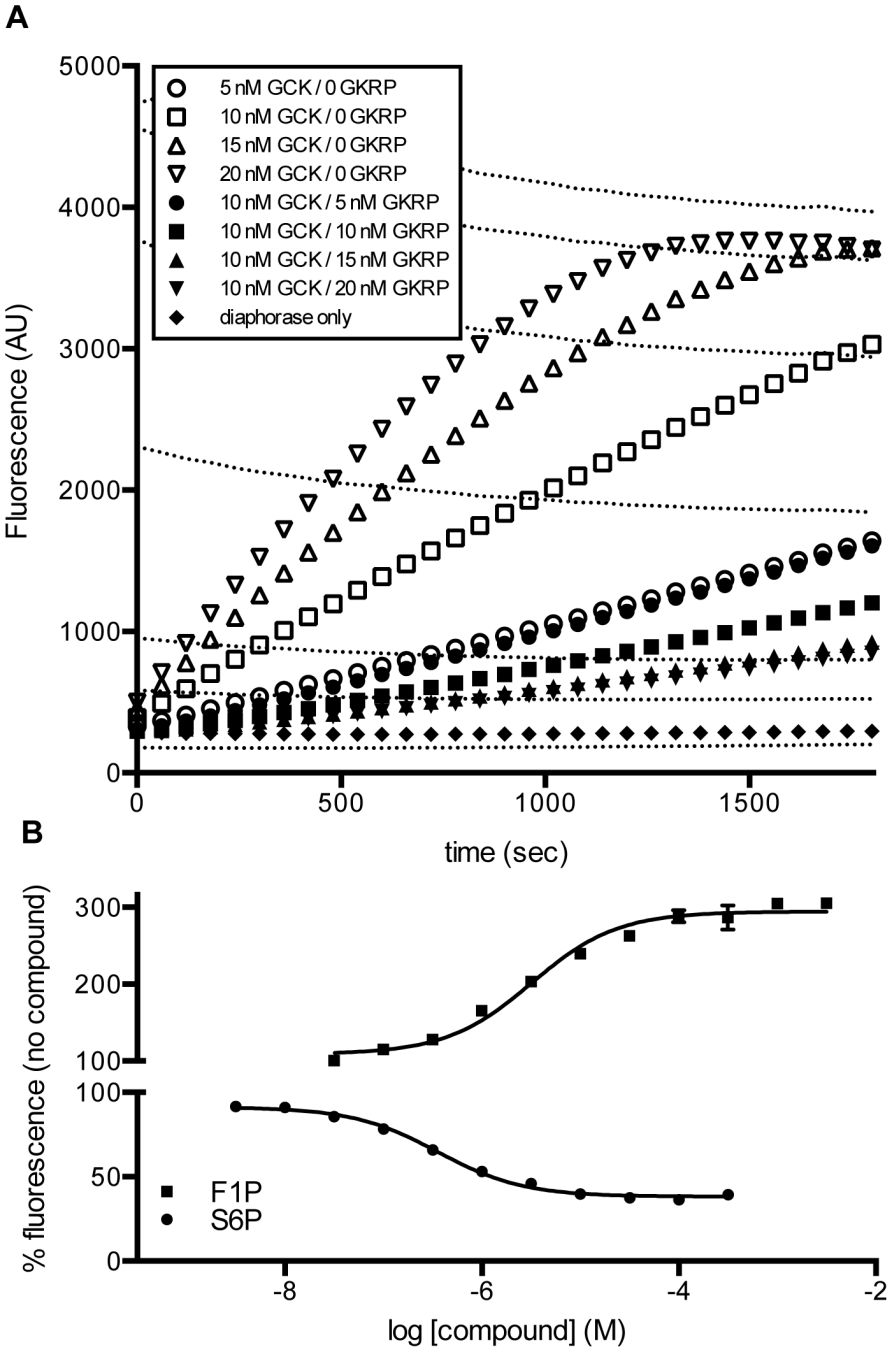
Optimization and validation of diaphorase-coupled detection of the GCK enzymatic reaction. (A) Reaction progress of the dual-coupled diaphorase reaction at various GCK and GKRP concentrations. Each data point is mean ± SEM for n = 8. Dashed lines are NADP^+^/NADPH controls, with the %NADPH increasing from the bottom (0%) to the top (100%) dashed line. (B) Effect of GKRP modulators on the dual-coupled diaphorase reaction. The reaction included 10 nM GCK and 10 nM GKRP. Results are shown for t = 10 minutes. Each data point is mean ± SEM for n = 2–4.

The G6PDH-coupled kinetic assay system described in [24] was utilized as a basis for developing a high-throughput screening assay to detect G6P generation. Because NADPH (excitation 340 nm, emission 450 nm) has spectral overlap with many fluorescent small molecule compounds, NADPH generation by G6PDH was coupled to NADPH-dependent reduction of resazurin to the fluorescent resorufin (excitation 525 nm, emission 590 nm) catalyzed by diaphorase [47] (**Figure 1B**). Initial assay validation was carried out over a range (0–20 nM) of GCK and GKRP concentrations (**Figure 4A** **)**. Additionally, seven different control mixes of NADP^+^ and NADPH were tested in duplicate to generate a standard curve. The total NADP^+^/NADPH concentration was 0.15 mM for all controls, with 0, 5, 10, 30, 50, 70, or 100% NADPH. This allowed estimation of the amount of NADP^+^ that had been converted by the GCK reaction throughout the time-course (**Figure 4A**, dashed lines). The assay is sensitive to very low levels of conversion.

We selected 10 nM GCK and 10 nM GKRP as providing a suitable window (81% inhibition of GCK at t = 5 minutes and 76% inhibition at t = 10 minutes, with fluorescence values equivalent to 5–10% NADPH generation) to detect the presence of GCK activators, which would be expected to enhance fluorescence, based on the difference between this curve and the curve at 10 nM GCK in the absence of GKRP. The reaction rate for both 10 nM GCK alone and for 10 nM GCK with 10 nM GKRP was linear for the entire 30-minute time-course. The combination of linearity and the relatively low percent conversion of NADP^+^ led us to select t = 10 minutes as providing an appropriate signal window for all future analyses. The response of 10 nM GCK and 10 nM GKRP to S6P and F1P using the diaphorase assay was then tested for 11 concentrations in quadruplicate (S6P IC_50_ = 0.4±0.04 µM; F1P EC_50_ = 4.0±0.5 µM; mean ± SD; **Figure 4B**). The IC_80_ value for S6P was calculated as 1.5 µM. Accordingly, this S6P concentration was utilized for all remaining diaphorase-coupled experiments.

Screening of the LOPAC^1280^ library was carried out in the presence of 10 nM GCK, 10 nM GKRP, and 1.5 µM S6P. Controls included GKA-EMD as for the HTRF assay, all assay components without GCK or GKRP (negative control; n = 16 wells/plate), and all assay components with 10 nM GCK only (uninhibited GCK, n = 16 wells/plate) to represent the maximum uninhibited signal. Compounds were pin-transferred into a 2 µl volume containing assay buffer, diaphorase, GCK, GKRP, and S6P. The plate reaction was initiated by addition of 2 µl substrate mix containing resazurin, G6PDH, ATP, glucose, and NADP^+^ in assay buffer and the plate was read immediately and every minute for 30 minutes. The 10-minute timepoint was used for analysis, yielding an excellent assay statistical performance (**Figure S6**; Z’ = 0.87±0.036; CV = 3.6±1.6; S/B = 33.8±6.7; mean ± SD for six plates).

### Bioluminescence-based Assay to Monitor the GCK Enzyme Reaction

The firefly luciferase enzyme utilizes luciferin and ATP to generate bioluminescence, and the coupling of ATP-dependent kinases to luciferase activity has enabled kinase activity to be measured quantitatively by luminescence measurements in high-throughput screens. The ADP-Glo™ assay, utilizing a two-step luciferase-based ADP-detection protocol, was employed to measure GCK activity (**Figure 1C**). Initial validation was carried out in 384-well plate format, and focused on GCK in the absence of GKRP to test suitability of the ADP-Glo™ format for the GCK enzyme. A titration of 14 GCK concentrations (1.5–100 nM; n = 8 for each concentration) was assessed at 5 mM glucose and 0.4 mM ATP at pH 7.4. The reaction was incubated for 45 minutes at room temperature. To estimate percent conversion of ATP to ADP, mixtures of ATP and ADP (total concentration 0.4 mM) were tested in duplicate, containing 0, 3, 5, 10, 30, 50, 70, and 100% ADP. The luminescence value at 10% ADP corresponded to the luminescence generated by 4 nM GCK (**Figure S7**). The ADP-Glo™ kit is linear throughout the range tested and provides an excellent range of detection.

**Figure 5:**
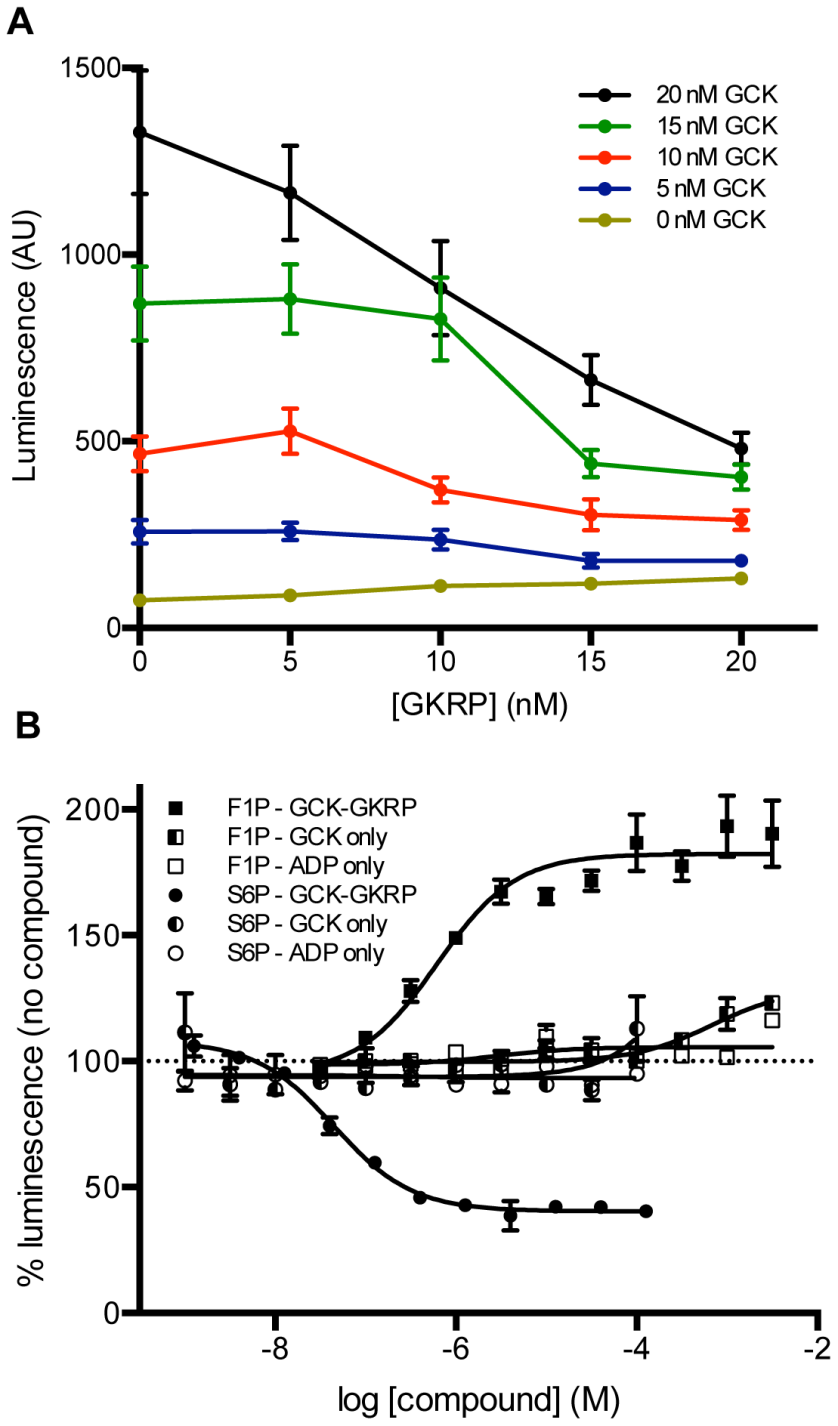
Optimization and validation of luminescence-based detection of the GCK enzymatic reaction. (A) The assay (pH 7.1) contained 5 mM glucose, 0.4 mM ATP, 2 µM S6P, and was terminated after 75 minutes. Each data point is mean ± SEM for n = 4. (B) Effect of GKRP modulators on the luminescence reaction. The reaction included 15 nM GCK and 15 nM GKRP (GCK-GKRP), 4 nM GCK (GCK only), or 0.04 mM ADP and no GCK or GKRP (ADP only). Assays were incubated for 75 minutes. Each data point is mean ± SEM for n = 2–4.

The effects of GKRP inhibition were then assessed using the same reaction conditions at pH 7.1. ADP generation was measured using a matrix of five concentrations each of GCK and GKRP (0, 5, 10, 15, and 20 nM) in quadruplicate (**Figure 5A**). Reactions were incubated for 75 minutes. Luminescence of 15 nM GCK was reduced by approximately 60% by 15 nM GKRP, and the mean S/B at 15 nM GCK +15 nM GKRP was 3.7.

Accordingly, the bioluminescence reaction was capable of measuring GCK enzymatic activity and its inhibition by GKRP. We therefore pursued miniaturization of the assay to 1536-well plate format and tested the effects of S6P and F1P in three different assay formats: 0.04 mM ADP in the absence of GCK and GKRP, 4 nM GCK, and 15 nM GCK with 15 nM GKRP (**Figure 5B**). All assays provided robust luminescence signal in the absence of F1P or S6P (S/B >4.0). As expected, response to F1P and S6P were dependent on the presence of GKRP (**Figure 5B**), with F1P activating the GCK enzymatic reaction (EC_50_ = 1±0.2 µM; mean ± SD) and resulting in an increase in luminescence signal, and S6P decreasing luminescence (IC_50_ = 0.005±0.0006 µM; mean ± SD).

Following these validations, reaction conditions of 15 nM GCK, 15 nM GKRP, and 2 µM S6P were selected for screening of the LOPAC^1280^ library at 0.4 mM ATP and 5 mM glucose. Compounds were added to assay mix containing all components except ATP, and the reaction was then initiated by ATP. Controls included GKA-EMD, all assay components without GCK but with 15 nM GKRP (negative control; n = 16 wells), and all assay components with 15 nM GCK only (uninhibited GCK; n = 16 wells) to represent the maximum uninhibited signal. The screening assay performed well (**Figure S8**; Z’ = 0.70±0.058; CV = 4.2±1.1; S/B = 4.2±0.1; mean ± SD for six plates).

### Cellular Analysis of GCK Translocation

**Figure 6:**
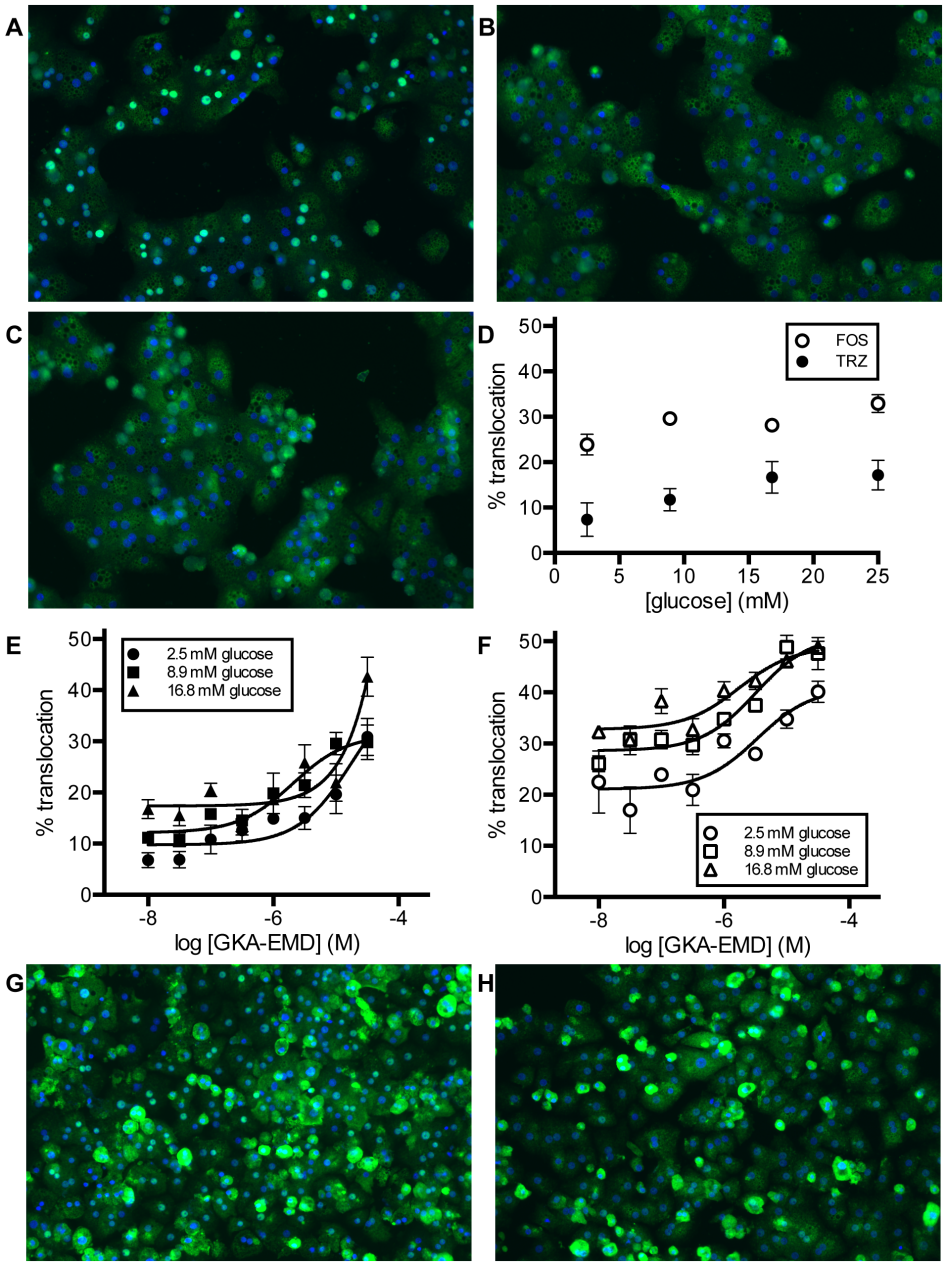
Localization of GCK in cryopreserved hepatocytes. Overlay of GCK (FITC channel; green) localization with Hoechst nuclear stain (blue) in cryopreserved hepatocytes from images collected at 10X magnification. The contrast was held fixed for all images, and the full dynamic range of all the 12 bit images has been maintained. (A–C) GCK localization in male Sprague-Dawley rat hepatocytes at (A) 2.5 mM glucose, (B) 16.7 mM glucose, and (C) 2.5 mM glucose and 31.6 µM GKA-EMD. (D–H) GCK localization in cryopreserved human hepatocytes. (D) Translocation of GCK in GKRP-positive cells in two separate lots (TRZ: closed symbols; FOS: open symbols) of human hepatocytes at various concentrations of glucose. (E–F) Translocation of GCK in the presence of GKA-EMD at various concentrations of glucose for donor TRZ and FOS, respectively. (G–H) Representative images from donor TRZ at (G) 2.5 mM glucose and (H) 2.5 mM glucose with 31.6 µM GKA. Data points are mean ± SEM for n = 4–8.

A number of previous studies have demonstrated high-content methodologies for analyzing GCK nuclear-to-cytoplasmic translocation in the presence of glucose, GKAs, and F1P precursors such as fructose and sorbitol in freshly isolated rat hepatocytes [30], [48], [49]. We investigated whether these protocols could be extended to cryopreserved pooled Sprague-Dawley rat hepatocytes. Twenty-four hours after plating in 96-well plates, hepatocytes were glucose-starved for 12 hours, and then treated with glucose or GKA-EMD for 2 hours before immunostaining for both GCK and GKRP localization. While GKRP predominantly localized to the nucleus in all conditions tested, we observed strong glucose-dependent GCK translocation (84% of cells with translocated GCK in the presence of 16.7 mM glucose, compared to 25% at 2.5 mM glucose; **Figure 6A–B**), as well as GKA-dependent GCK translocation (83% translocation at 2.5 mM glucose in the presence of 31.6 µM GKA; **Figure 6C**), suggesting cryopreserved hepatocytes can be utilized to model GCK translocation.

Previous attempts to develop human cellular models of GCK translocation, either using endogenous proteins in Hep G2 or HepaRG cells or generating overexpression systems by transfection of human *GCK* and/or *GCKR* in HeLa cells or primary mouse hepatocytes, have failed to reproduce results observed in rodent models [3], [12]. We therefore investigated whether cryopreserved human hepatocytes from two different donors, without known T2D, dyslipidemia, or drug use, could be utilized as models for GCK translocation. In all conditions tested, there was a significant sub-population of GCK-positive cells with complete cytoplasmic localization that were negative for GKRP. Accordingly, to detect translocation, we developed an analysis methodology to restrict quantitation of GCK translocation to GKRP-positive cells. The basal nuclear localization of GCK from lot FOS was approximately three-fold higher than lot TRZ (**Figure 6D**). Both lots showed modest translocation in response to glucose, with the number of translocation-positive cells approximately 10% greater at 25 mM glucose compared to 2.5 mM glucose. However, even at 25 mM glucose, the percentage of translocation-positive cells from lot TRZ was lower than the percentage from lot FOS under any condition tested. Lot FOS showed some evidence of translocation in response to GKA (approximately two-fold at maximum; EC_50_ = 3.4±2.0, 3.2±1.4, and 1.7±0.9 µM at 2.5, 8.9, and 16.8 mM glucose, respectively; mean ± SD; **Figure 6F**), but no translocation was observed in the presence of sorbitol. Lot TRZ demonstrated greater translocation in response to sorbitol (approximately four-fold at 2.5 mM glucose) and GKA-EMD (approximately 4.5-fold at 2.5 mM glucose) (**Figure 6E, G–H**). Interestingly, when the same glucose starvation and compound treatment protocol was utilized for freshly isolated human hepatocytes from a single obese female donor, GKRP expression was undetectable across all conditions tested (including control wells that were not glucose starved or compound treated), and GCK localization was exclusively cytoplasmic (**Figure S9**). While these results are preliminary, significant heterogeneity of GCK localization between individuals is consistent with previous reports of variation in human GCK localization and activity due to factors including genetic variation in *GCKR* and metabolic conditions such as obesity or T2D [3], [12], [50], [51].

## Discussion

During the past decade, human genetics studies have emphasized the critical importance of both GCK and GKRP in health and disease. Additionally, significant kinetic and structural efforts have greatly increased understanding of the properties of the interaction between GCK and GKRP, particularly in the context of pharmacological modulation of GCK. However, to date, small molecule screening efforts have primarily focused on GCK enzymatic activity, with testing of GKRP inhibition of GCK restricted to secondary validation experiments [14]. We focused primarily on GKRP, a potentially attractive target because of its unique tissue specificity, distinct regulation from GCK, and lack of known small molecule probes other than physiological regulators such as F1P and S6P. Here, we describe three unique assays to measure the interaction between recombinant human GCK and GKRP that could be further used to characterize small molecules of interest, as we demonstrate for S6P, F1P, and previously described GKAs. All three GCK-GKRP assays developed were robust in 1536-well format, indicating they could be useful for further small-molecule high-throughput screens or as validation assays. In our screens of the LOPAC^1280^ library, we included S6P at its EC_80_ value in all assays to bias us towards detecting potential competitors of S6P; however, each assay can also be performed in the absence of S6P. The overall hit rate was very low for these three assays (3 to 4 hits per assay representing a hit rate of ∼0.2%), which is consistent with a recent publication by Amgen in a GCK-GKRP screen in which two weak hits were identified from the library screen and subsequently optimized [52], [53]. Suramin and ATA were identified as inhibitors of the GCK-GKRP interaction in all three assays described herein. These compounds, however, are known to be promiscuous and were not pursued further.

This orthogonal set of assays detects luciferase-driven luminescence, fluorescence emission at 590 nm, or time-resolved fluorescence with emission at 615 and 665 nm, minimizing detection modality overlap and the potential for false positives. Each assay type also has additional features that may be useful for specific applications. For example, the diaphorase-coupled and ADP-Glo™ assays were also validated and optimized in the absence of GKRP, demonstrating that these assays could be utilized in mechanistic investigations, potentially distinguishing the mode of action of small molecules for activity against GKRP itself, GCK, or the interaction of the two proteins. As both the coupling enzymes and the detection modalities (luminescence vs. fluorescence) differ between the ADP-Glo™ and diaphorase-coupled assays, it is unlikely that a compound would interfere with both assay types. Additional differences between the ADP-Glo™ and diaphorase-coupled assays include that the former is run in end-point mode while the latter is run in kinetic mode and that the cost of the diaphorase-coupled assay is lower. The HTRF assay was notable in that it required a relatively small amount of GKRP, with the final optimized assay including 2.5 nM GKRP in a 4 µl volume. This is an important feature in light of the known challenges in producing large quantities of recombinant human GKRP [24], [34]. Additionally, relevant to the emerging differences in the glucose-dependent kinetic behavior of GCK with respect to classical GKAs, the HTRF assay can be run in either the absence or presence of glucose [28], [30]. Finally, we note that the IgG-based counter-assay developed for the HTRF assay could be generally applied to other HTRF-based screens as a validation step in future follow-up screens of putative HTRF screening hits.

The nuclear-to-cytoplasmic translocation of rodent GCK in response to glucose and small molecule modulators such as GKAs has been extensively validated [3], [48], [49]. However, efforts to develop a robust and reproducible translocation system for human GCK and GKRP in non-primary cell types, such as HeLa cells, Hep G2 cells, and HepaRG cells, have not met with the same degree of success [3], [12]. We therefore explored the possibility of extending previously described cellular assays from freshly isolated rat hepatocytes to cryopreserved rat and human hepatocytes. The presence of GKRP and GCK was confirmed in cryopreserved rat hepatocytes, and our results were consistent with previous findings in freshly isolated rat hepatocytes, with near-complete cytoplasmic translocation of rat GCK observed at high glucose and/or GKA concentrations (**Figure 6A–C**). Immunofluorescence of both GCK and GKRP in cryopreserved human hepatocytes could also be detected, but expression of GKRP was undetectable in a significant sub-population of cells. Accordingly, translocation of GCK was analyzed in cells positive for both proteins, demonstrating the importance of measuring GCK and GKRP simultaneously and the necessity for careful analysis to interpret collected images. Notably, the extent of GKRP-positive cells, and therefore the subcellular localization of GCK, differed across donors. These findings are consistent with reports of variation in GCK and GKRP localization due to metabolic dysfunction, genetic variation, and dietary factors, and may warrant further comprehensive characterization [3], [50], [51], [54].

The total extent of GCK translocation observed in GKRP-positive human hepatocytes was not as complete as was seen in rat hepatocytes, with a maximal observed cytoplasmic translocation of 43% GKRP-positive cells, compared to 84% in rat. Although the diminished extent of translocation observed here may be in part due to the need to find optimized human culture conditions, the decreased translocation observed is consistent with results comparing human and rat GKRPs, either expressed recombinantly or transiently transfected into mouse hepatocytes [3], [34]: human GKRP is a more potent inhibitor of GCK than its rodent ortholog, localizes more strongly to the nucleus, and sequesters GCK to a greater extent [3]. Our results therefore suggest that rodent models of GCK translocation may be insufficient to understand fully the cellular effects of human hepatic GCK activation by glucose or other small-molecule activators. This assay can be used to test activators in a cellular context, and this work extends the previous work in a rat line into a human line.

In summary, we have developed a diverse toolkit to query the interaction of human GCK and GKRP. The assays have a number of potential uses, including high-throughput small molecule screening to discover novel modulators, as well as mechanistic studies. For instance, the HTRF assay could be utilized to investigate differences in the properties of variant forms of GCK and/or GKRP relevant to human disease, or in future structural or biophysical studies, particularly in light of the recent crystal structures of GKRP and the GCK-GKRP complex [26], [27]. We also provide further support for the existence of important cellular differences between rodent and human GCK and GKRP, which may inform future work on more relevant primary models of human health or disease states, or the development of other model cellular systems such as iPS-derived hepatocytes.

## Supporting Information

Figure S1 **Calculation of GCK glucose S_0.5_ using a luciferase-based bioluminescence assay.** The assay included 4 nM GCK and 4 mM ATP and was terminated after 45 minutes. Each data point is mean ± SEM (n = 8). Curves were fit using nonlinear regression with the allosteric sigmoidal option in GraphPad Prism to determine the S_0.5_ and Hill coefficient.
(TIF)

Figure S2 **Calculation of the GCK ATP K_m_ by following NADH oxidation with a PK/LDH dual-coupled system.** (A) Reaction scheme of PK/LDH dual-coupled assay. The reaction included 4 nM GCK. (B) Reaction progress over the first 60 seconds was monitored by the loss of NADH fluorescence at 450 nm using a ViewLux Microplate Imager and results analyzed by linear regression. Resultant slopes from regression were plotted and the curve fit using nonlinear regression accounting for substrate inhibition in GraphPad Prism to give a best-fit K_m_ value.
(TIF)

Figure S3 **Analysis of coupling components in the diaphorase-coupled enzyme system.** Experiments were run in the absence of GCK and GKRP. (A) NADPH-dependence of 0.1 mg/ml diaphorase activity in the presence of 0.1 mM resazurin, monitored by the generation of resorufin. (B) G6P-dependence of 4 U/ml G6PDH activity in the presence of 0.15 mM NADP^+^ and in the absence of diaphorase and resazurin. NADPH generation was monitored fluorescently using a ViewLux Microplate Imager. Each data point is mean ± SEM for n = 2. Lines were fit using linear regression (r^2^ = 0.99 and 0.98, respectively; GraphPad Prism) excluding the highest concentrations, which are shown to indicate that maximal enzymatic conversion (i.e., substrate limitation) has occurred.
(TIF)

Figure S4 **Time dependence of GCK-GKRP HTRF signal.** Fluorescence at both the fluorescence donor wavelength (615 nm) and fluorescence acceptor wavelength (665 nm) was measured every 30 minutes for 240 minutes. The assay was incubated at room temperature in the dark throughout the course of the experiment.
(TIF)

Figure S5 **Comparison of selected compounds in the GCK-GKRP HTRF assay and IgG HTRF counter-assay.** Reaction conditions for the IgG counter-assay were identical to those in the GCK-GKRP HTRF assay excluding GCK and GKRP, with the exception of replacement of 0.0945 ng/well anti-GST K with 0.0945 ng/well of anti-mouse IgG. All graphs are presented relative to the HTRF ratio in the absence of compound (100%). (A) S6P and F1P did not affect the signal of the IgG counter-assay. The curves for F1P and S6P in the GCK-GKRP assay are from Figure 2; each data point for the IgG assay is mean ± SEM for n = 2. (B–C) Two selected compounds from the LOPAC^1280^ library, suramin sodium salt and aurintricarboxylic acid (ATA), which scored as potential hits in the primary screening, were selected to demonstrate the potential utility of the IgG counter-assay. (B) Suramin sodium salt showed autofluorescence in the pre-read measured at 665 nm, and also reduced the FRET signal in the IgG counter-assay in a concentration-dependent manner. (C) ATA was not autofluorescent at either the acceptor or donor wavelength, but reduced the FRET signal in the IgG counter-assay in a concentration-dependent manner.
(TIF)

Figure S6 **Quality metrics for the diaphorase assay with the LOPAC^1280^ library in 1536-well plates.** (A) Z’ factor, (B) signal/background, and (C) % variance as a function of assay plate.
(TIF)

Figure S7 **GCK dependence of luciferase-based bioluminescence assay.** The reaction included GCK, no GKRP, 5 mM glucose, 0.4 mM ATP and was terminated after 45 minutes. Points are plotted as estimated %ATP converted by GCK by comparison to control mixtures of ATP and ADP. Each data point is mean ± SEM for n = 4.
(TIF)

Figure S8 **Quality metrics for the bioluminescence assay with the LOPAC^1280^ library in 1536-well plates.** (A) Z’ factor, (B) signal/background, and (C) % variance as a function of assay plate.
(TIF)

Figure S9 **GCK localization in a single batch of freshly-isolated human female hepatocytes.** An overlay of GCK (FITC channel; green) localization with Hoechst nuclear stain (blue) is shown from images collected at 10X magnification. Culture conditions, compound treatment, and imaging methodology were identical to those utilized for cryopreserved human hepatocytes. There was no signal detectable in the Cy5 (GKRP) channel.
(TIF)

Table S1 **Protocol Sequence for GCK-GKRP HTRF qHTS Assay.**
(TIF)

Table S2 **Protocol Sequence for GCK-GKRP Diaphorase qHTS Assay.**
(TIF)

Table S3 **Protocol Sequence for GCK-GKRP Bioluminescence qHTS Assay.**
(TIF)
