# Supplementary figures and images for: A Panel of Diverse Assays to Interrogate the Interaction between Glucokinase and Glucokinase Regulatory Protein, Two Vital Proteins in Human Disease
Source: PLoS One. 2014 Feb 19;9(2):e89335. doi: 10.1371/journal.pone.0089335 (PMC3929664; doi:10.1371/journal.pone.0089335)

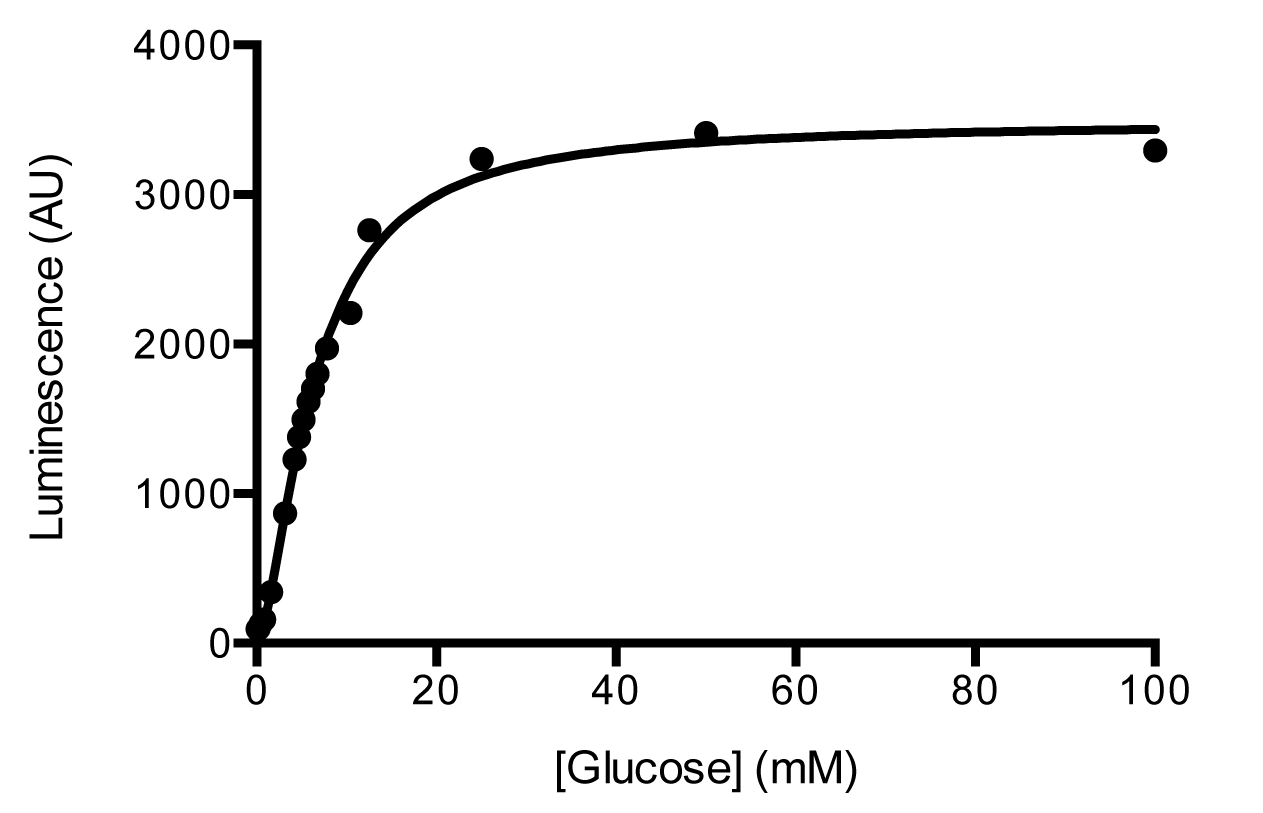

Supplement: Figure S1 — Calculation of GCK glucose S0.5 using a luciferase-based bioluminescence assay. The assay included 4 nM GCK and 4 mM ATP and was terminated after 45 minutes. Each data point is mean ± SEM (n = 8). Curves were fit using nonlinear regression with the allosteric sigmoidal option in GraphPad Prism to determine the S0.5 and Hill coefficient. (TIF) [file pone.0089335.s001.tif]

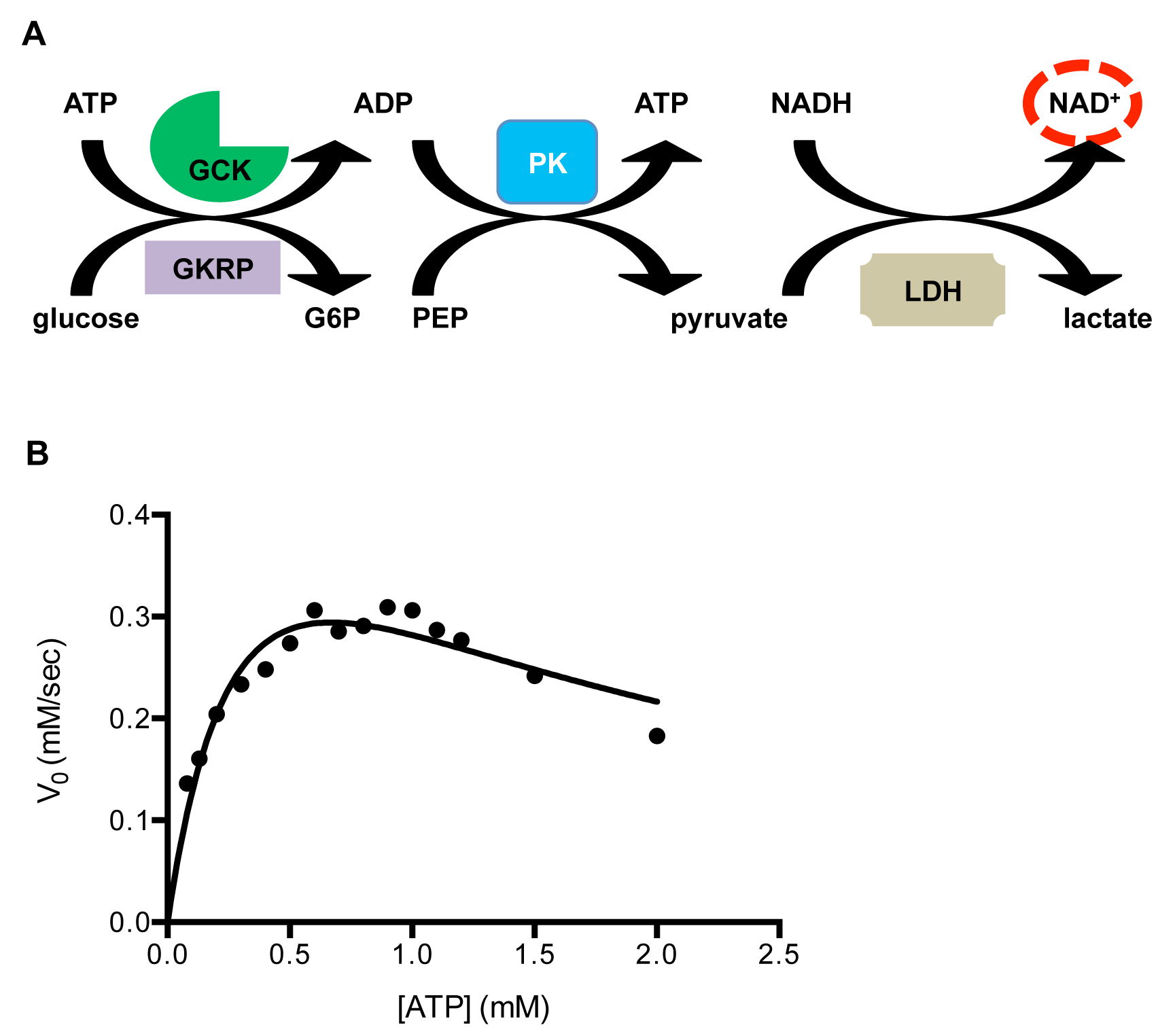

Supplement: Figure S2 — Calculation of the GCK ATP Km by following NADH oxidation with a PK/LDH dual-coupled system. (A) Reaction scheme of PK/LDH dual-coupled assay. The reaction included 4 nM GCK. (B) Reaction progress over the first 60 seconds was monitored by the loss of NADH fluorescence at 450 nm using a ViewLux Microplate Imager and results analyzed by linear regression. Resultant slopes from regression were plotted and the curve fit using nonlinear regression accounting for substrate inhibition in GraphPad Prism to give a best-fit Km value. (TIF) [file pone.0089335.s002.tif]

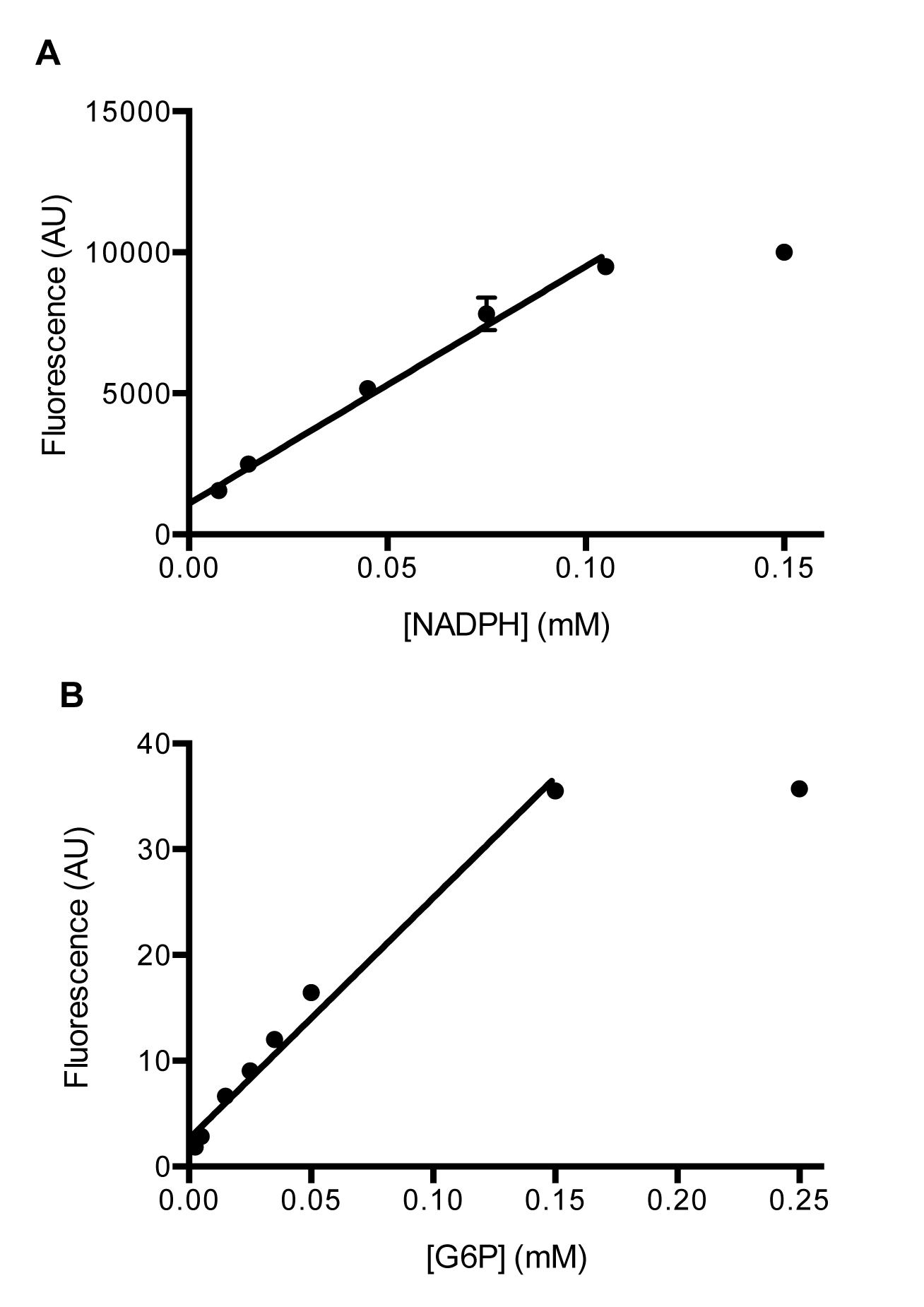

Supplement: Figure S3 — Analysis of coupling components in the diaphorase-coupled enzyme system. Experiments were run in the absence of GCK and GKRP. (A) NADPH-dependence of 0.1 mg/ml diaphorase activity in the presence of 0.1 mM resazurin, monitored by the generation of resorufin. (B) G6P-dependence of 4 U/ml G6PDH activity in the presence of 0.15 mM NADP+ and in the absence of diaphorase and resazurin. NADPH generation was monitored fluorescently using a ViewLux Microplate Imager. Each data point is mean ± SEM for n = 2. Lines were fit using linear regression (r2 = 0.99 and 0.98, respectively; GraphPad Prism) excluding the highest concentrations, which are shown to indicate that maximal enzymatic conversion (i.e., substrate limitation) has occurred. (TIF) [file pone.0089335.s003.tif]

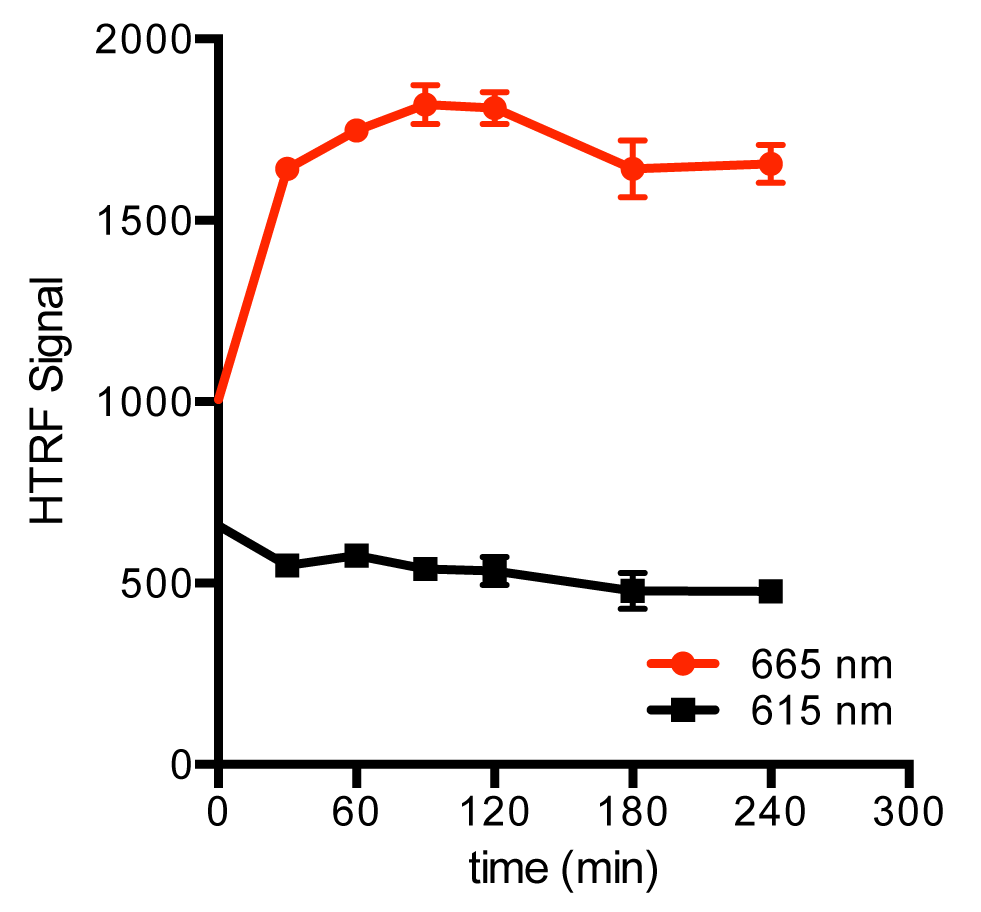

Supplement: Figure S4 — Time dependence of GCK-GKRP HTRF signal. Fluorescence at both the fluorescence donor wavelength (615 nm) and fluorescence acceptor wavelength (665 nm) was measured every 30 minutes for 240 minutes. The assay was incubated at room temperature in the dark throughout the course of the experiment. (TIF) [file pone.0089335.s004.tif]

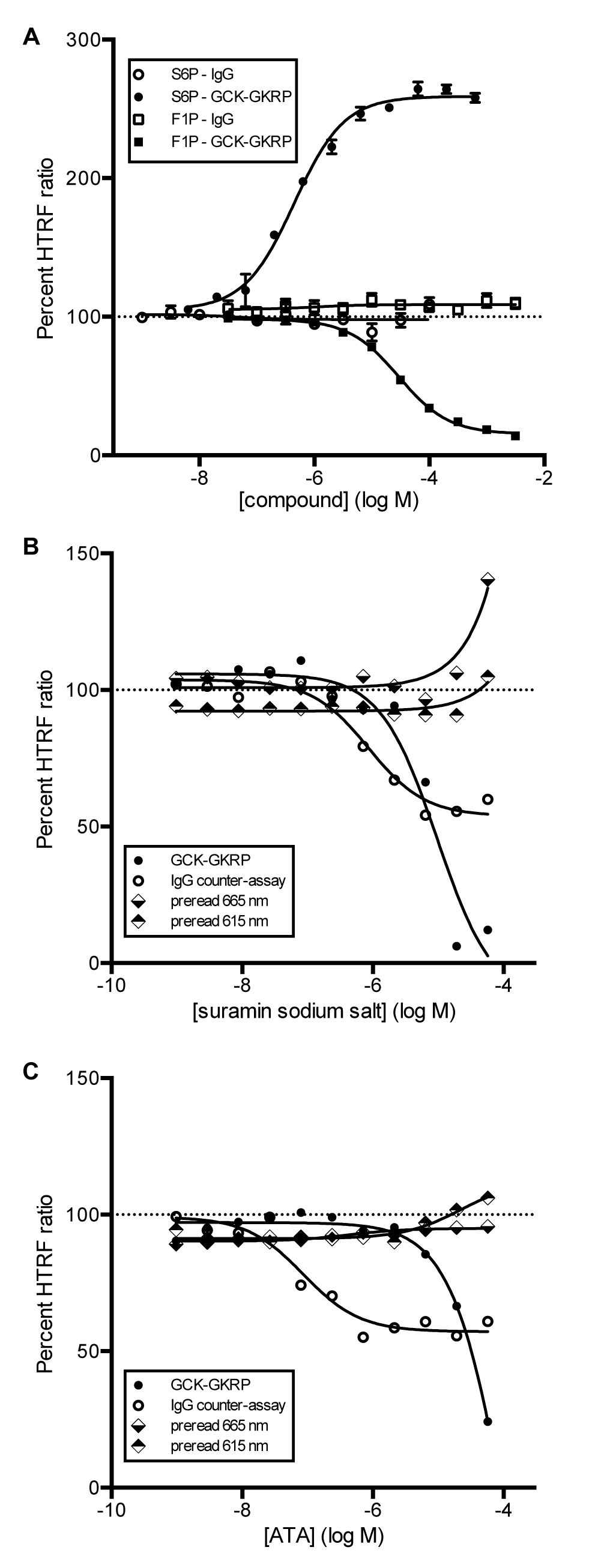

Supplement: Figure S5 — Comparison of selected compounds in the GCK-GKRP HTRF assay and IgG HTRF counter-assay. Reaction conditions for the IgG counter-assay were identical to those in the GCK-GKRP HTRF assay excluding GCK and GKRP, with the exception of replacement of 0.0945 ng/well anti-GST K with 0.0945 ng/well of anti-mouse IgG. All graphs are presented relative to the HTRF ratio in the absence of compound (100%). (A) S6P and F1P did not affect the signal of the IgG counter-assay. The curves for F1P and S6P in the GCK-GKRP assay are from Figure 2; each data point for the IgG assay is mean ± SEM for n = 2. (B–C) Two selected compounds from the LOPAC1280 library, suramin sodium salt and aurintricarboxylic acid (ATA), which scored as potential hits in the primary screening, were selected to demonstrate the potential utility of the IgG counter-assay. (B) Suramin sodium salt showed autofluorescence in the pre-read measured at 665 nm, and also reduced the FRET signal in the IgG counter-assay in a concentration-dependent manner. (C) ATA was not autofluorescent at either the acceptor or donor wavelength, but reduced the FRET signal in the IgG counter-assay in a concentration-dependent manner. (TIF) [file pone.0089335.s005.tif]

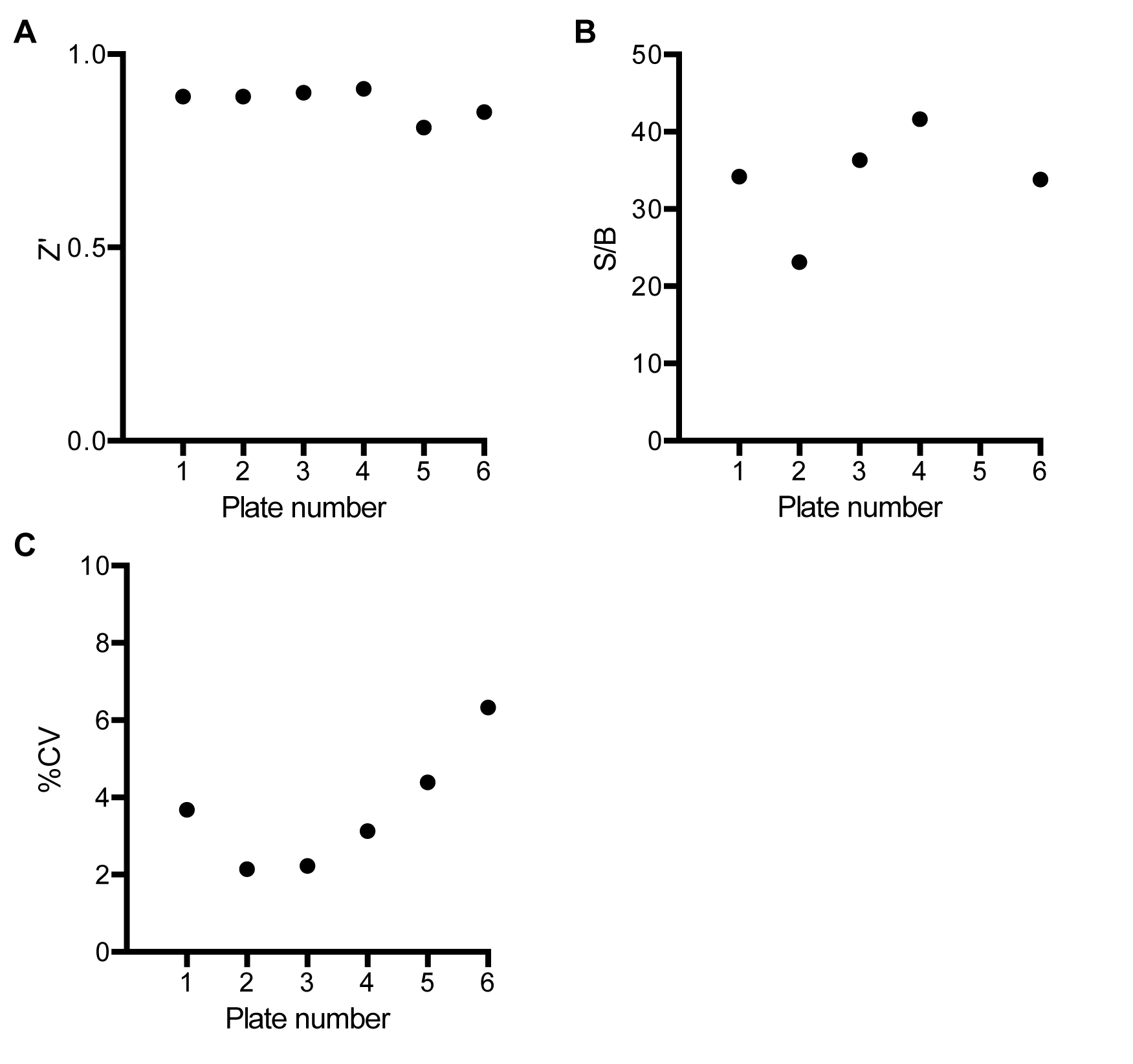

Supplement: Figure S6 — Quality metrics for the diaphorase assay with the LOPAC1280 library in 1536-well plates. (A) Z’ factor, (B) signal/background, and (C) % variance as a function of assay plate. (TIF) [file pone.0089335.s006.tif]

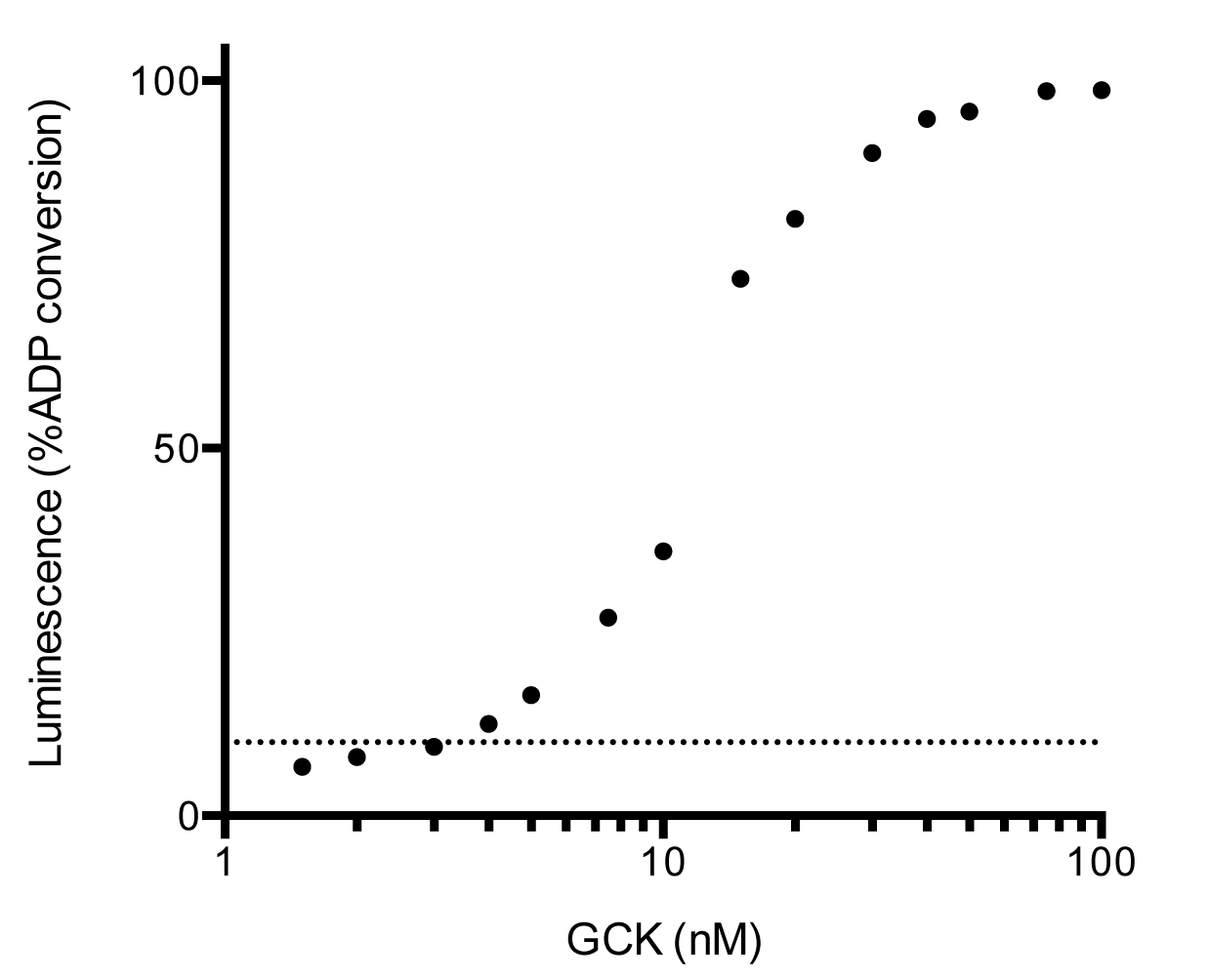

Supplement: Figure S7 — GCK dependence of luciferase-based bioluminescence assay. The reaction included GCK, no GKRP, 5 mM glucose, 0.4 mM ATP and was terminated after 45 minutes. Points are plotted as estimated %ATP converted by GCK by comparison to control mixtures of ATP and ADP. Each data point is mean ± SEM for n = 4. (TIF) [file pone.0089335.s007.tif]

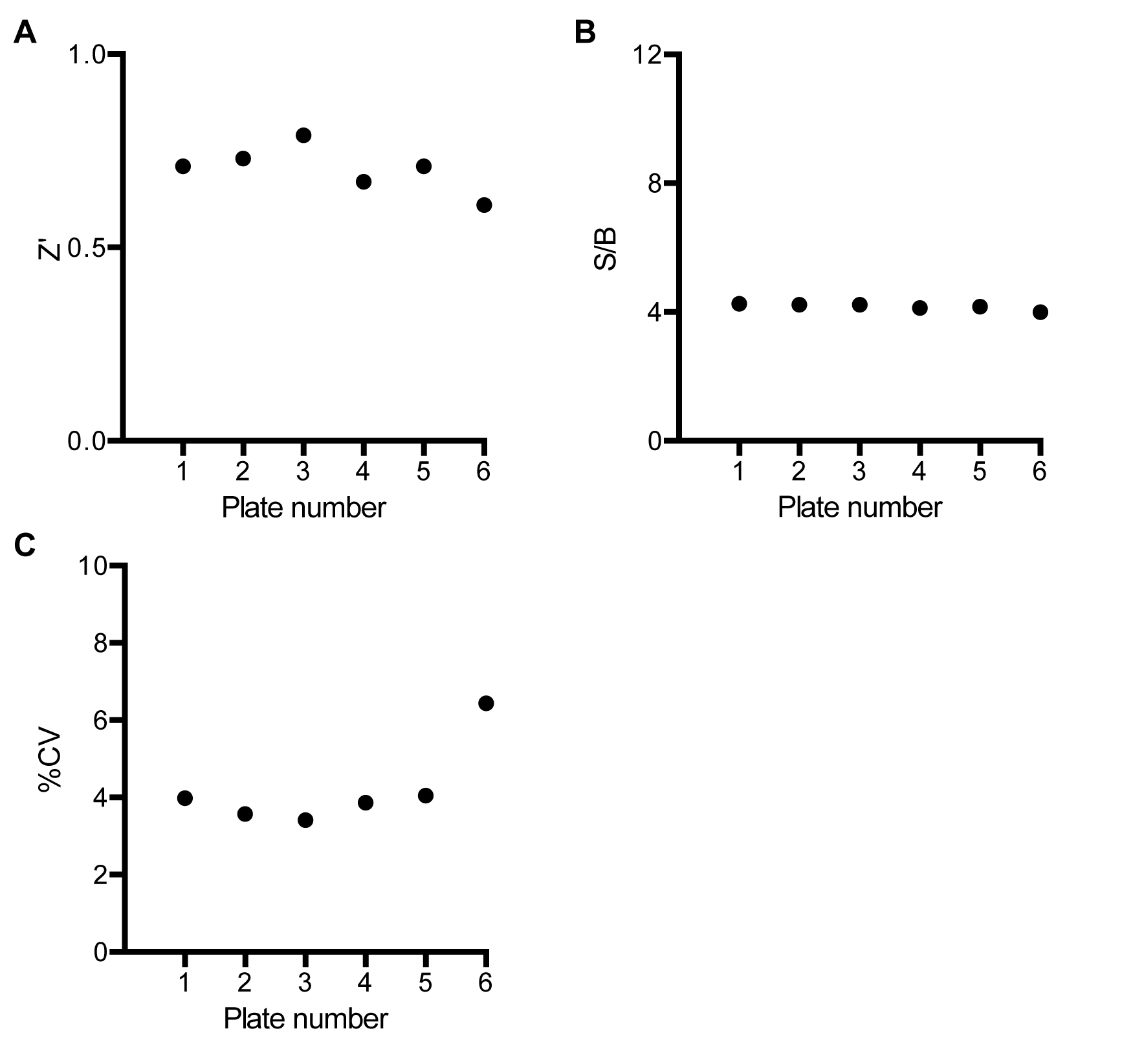

Supplement: Figure S8 — Quality metrics for the bioluminescence assay with the LOPAC1280 library in 1536-well plates. (A) Z’ factor, (B) signal/background, and (C) % variance as a function of assay plate. (TIF) [file pone.0089335.s008.tif]

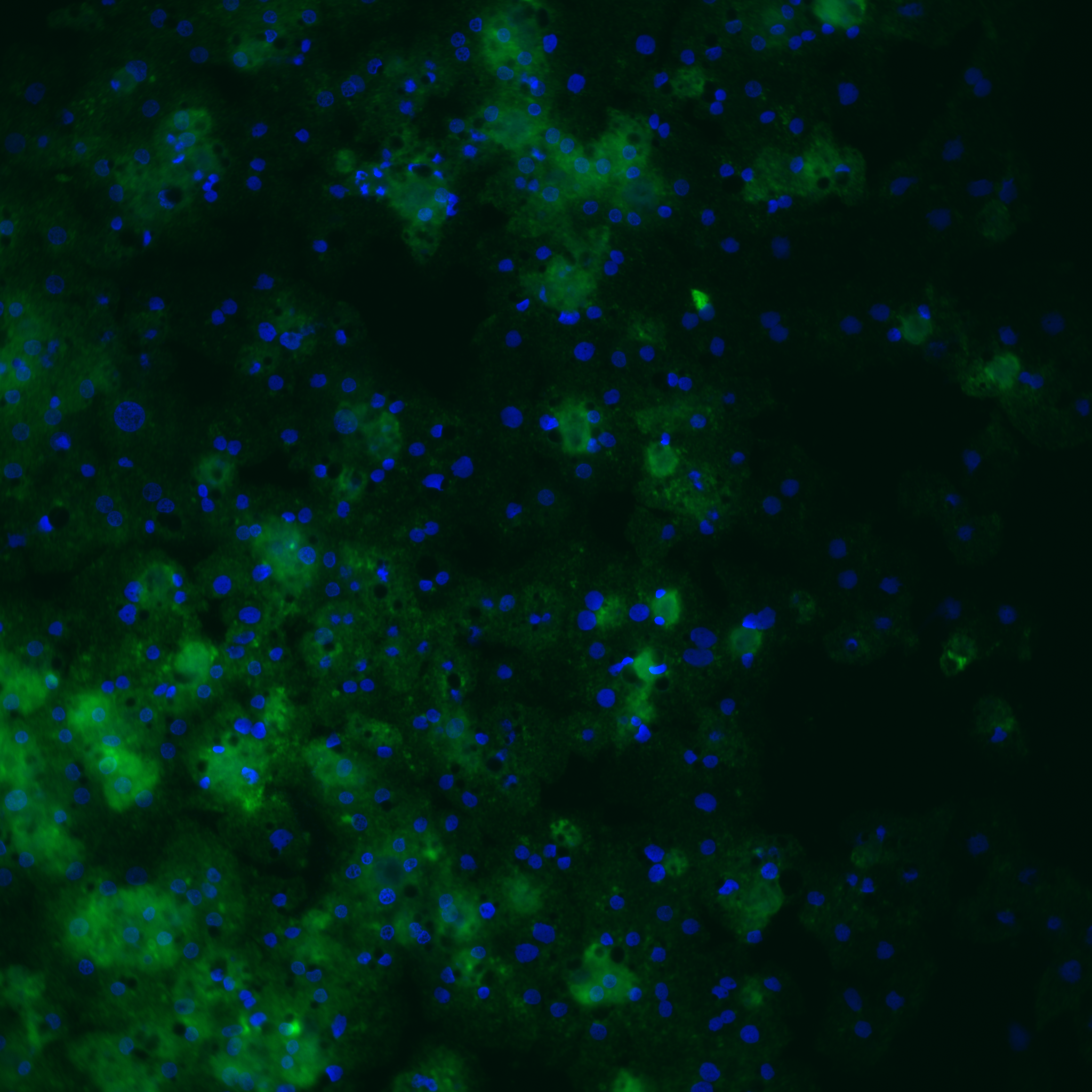

Supplement: Figure S9 — GCK localization in a single batch of freshly-isolated human female hepatocytes. An overlay of GCK (FITC channel; green) localization with Hoechst nuclear stain (blue) is shown from images collected at 10X magnification. Culture conditions, compound treatment, and imaging methodology were identical to those utilized for cryopreserved human hepatocytes. There was no signal detectable in the Cy5 (GKRP) channel. (TIF) [file pone.0089335.s009.tif]

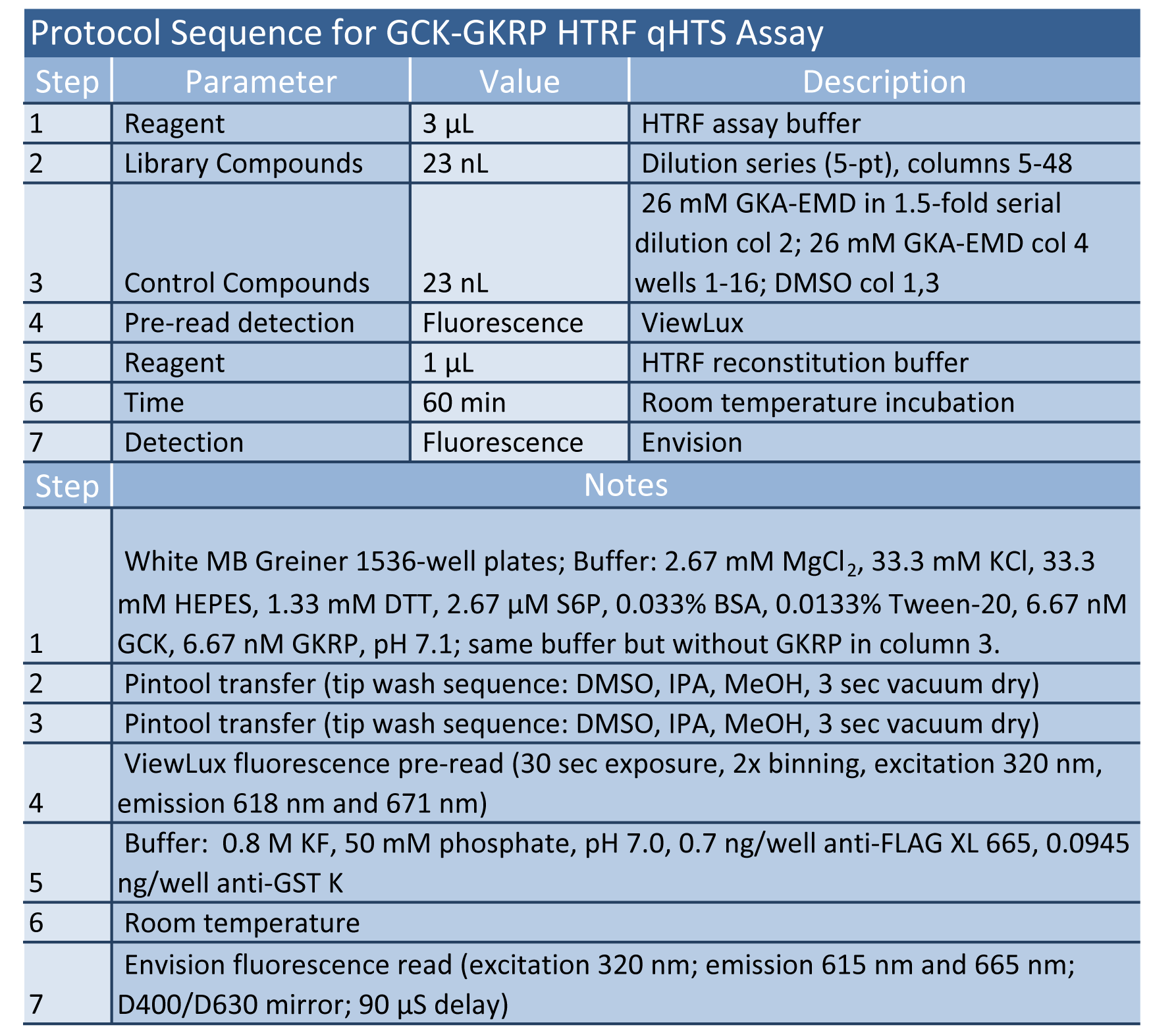

Supplement: Table S1 — Protocol Sequence for GCK-GKRP HTRF qHTS Assay. (TIF) [file pone.0089335.s010.tif]

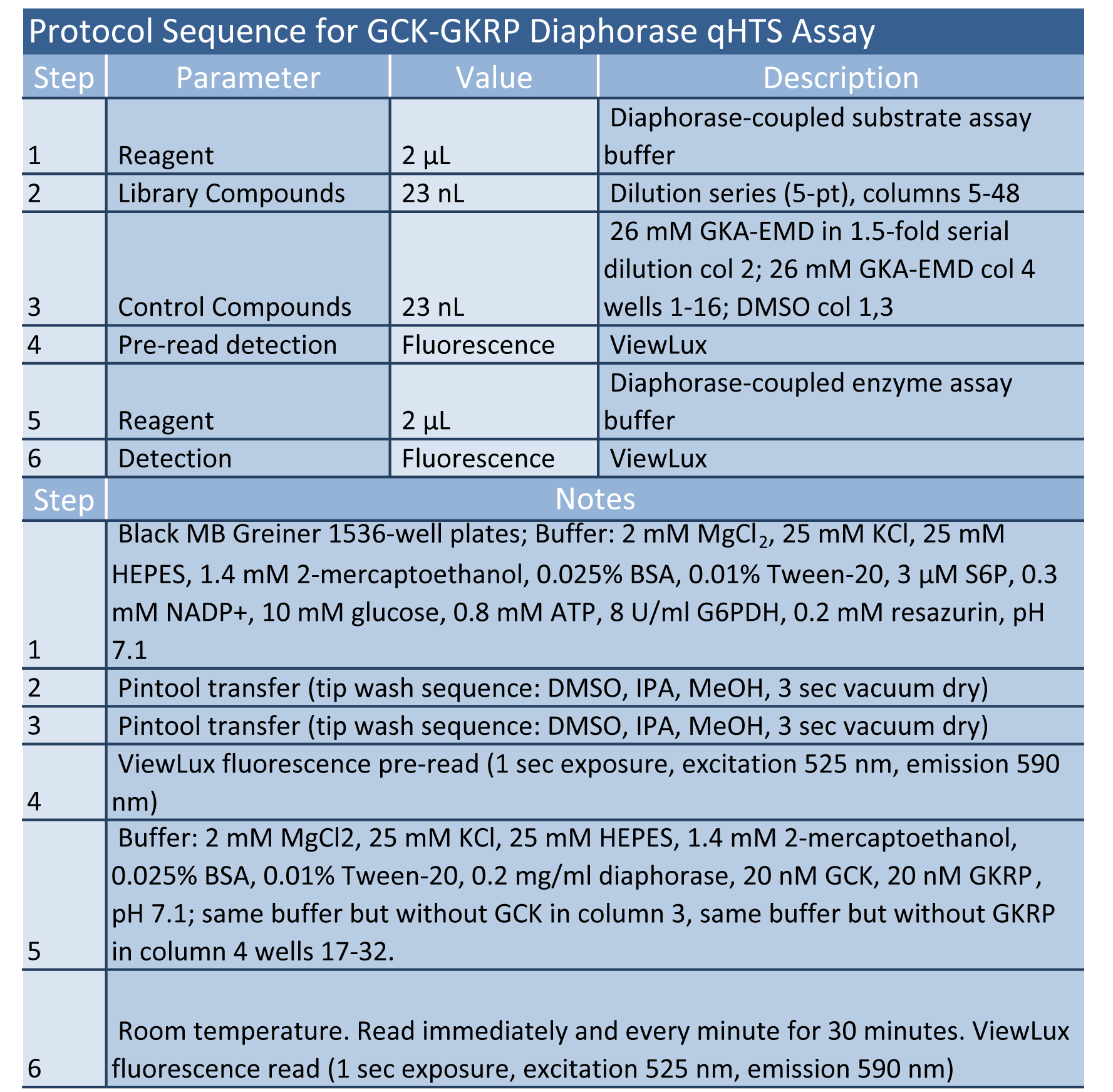

Supplement: Table S2 — Protocol Sequence for GCK-GKRP Diaphorase qHTS Assay. (TIF) [file pone.0089335.s011.tif]

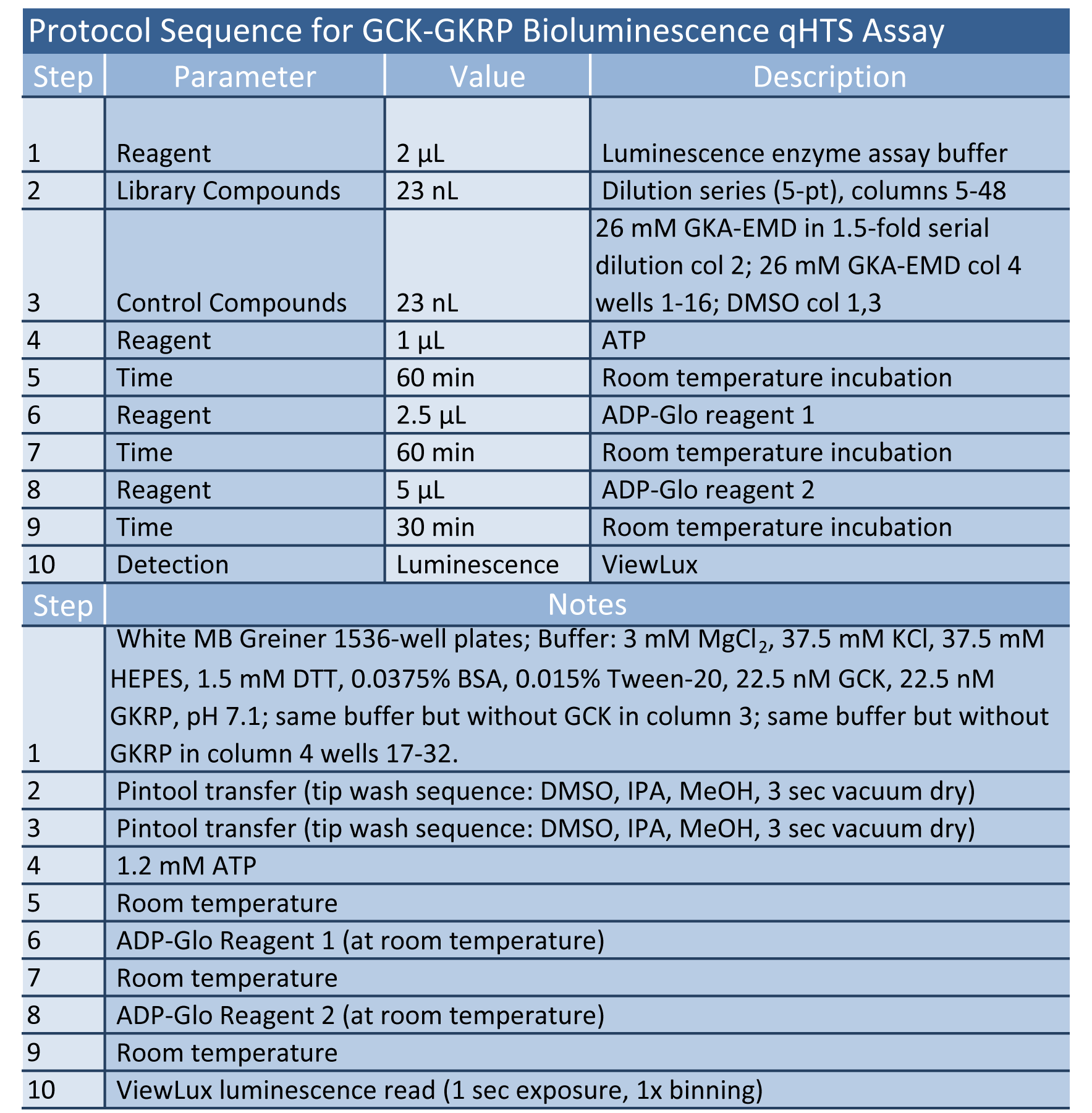

Supplement: Table S3 — Protocol Sequence for GCK-GKRP Bioluminescence qHTS Assay. (TIF) [file pone.0089335.s012.tif]
